# Small and Large Extracellular Vesicles in Circulation of Diffuse Large B‐Cell Lymphoma Patients Originate From Different Cell Types of the Tumor Microenvironment

**DOI:** 10.1002/jev2.70259

**Published:** 2026-03-26

**Authors:** Filippo Maltoni, Steven Wang, Mischa. F. B. Steketee, Cristina A. Gómez‐Martín, Esther E. E. Drees, Federica Morelli, Leontien Bosch, Monique van Eijndhoven, Gert Jan Timmers, Ilse Houtenbos, Josée M. Zijlstra, Xiaofei Ye, Qiang Pan‐Hammarström, Martine E. D. Chamuleau, Pier Luigi Zinzani, Yongsoo Kim, Lucia Catani, Dirk Michiel Pegtel

**Affiliations:** ^1^ Department of Surgical and Medical Sciences Institute of Hematology “L. e A. Seràgnoli”, University of Bologna Bologna Italy; ^2^ IRCCS Azienda Ospedaliero‐Universitaria di Bologna, Institute of Hematology “L. e A. Seràgnoli” Bologna Italy; ^3^ Department of Hematology Amsterdam UMC Location Vrije Universiteit Amsterdam Amsterdam the Netherlands; ^4^ Department of Pathology Amsterdam UMC Location Vrije Universiteit Amsterdam Amsterdam the Netherlands; ^5^ Cancer Center Amsterdam, Imaging and Biomarkers Amsterdam the Netherlands; ^6^ Amstelland Ziekenhuis Amstelveen the Netherlands; ^7^ Spaarne Gasthuis Haarlem the Netherlands; ^8^ Department of Biosciences and Nutrition Karolinska Institutet Stockholm Sweden; ^9^ Division of Immunology, Department of Medical Biochemistry and Biophysics Karolinska Institutet Stockholm Sweden

**Keywords:** extracellular vesicles, small and large EVs, diffuse large B‐cell lymphoma, liquid biopsy, miRNA, mRNA, deconvolution

## Abstract

Malignant and non‐malignant cells within the tumor microenvironment (TME) actively secrete extracellular vesicles (EVs) that may mediate intercellular communication or enter the blood stream. Circulating EVs in Diffuse Large B‐Cell Lymphoma (DLBCL) patients are a promising source of liquid biopsy biomarkers; however, whether different cellular components of the TME preferentially secrete small (S‐) and/or large (L‐) EVs is still unknown.

With an established density‐gradient separation protocol and tunable resistive pulse sensing analysis, we demonstrate that DLBCL cells in culture produce 100‐1000‐fold higher numbers of S‐EVs (50–200 nm) compared with L‐EVs (200–1000 nm) and very large EVs (>1000 nm). In contrast, the plasma from DLBCL patients contains comparable concentrations of S‐ and L‐EVs, consistent with various cellular origins. Small RNA sequencing showed minor differences in miRNA content between plasma S‐ and L‐EVs; however, messenger RNA sequencing revealed stark differences in cargo between EV‐size subtypes and between healthy donors and patients. Deconvolution analysis with single‐cell sequencing data from 17 DLBCL tumor tissues as reference using the Statescope algorithm indicated that circulating S‐EVs from malignant cells outnumber the L‐EVs. In contrast, TME macrophage‐, T cell‐, and natural killer‐derived L‐EVs outnumber S‐EVs. Together, these findings suggest that circulating S‐ and L‐EVs can originate from distinct cellular compartments within the DLBCL TME, representing complementary biological information. These observations have important implications for the development of EV‐based liquid biopsy strategies.

## 1 Introduction

Extracellular vesicles (EVs) are lipid bilayer‐enclosed particles released by nearly all cell types (Van Niel et al., 2018). These vesicles carry a heterogeneous bioactive cargo, including proteins, lipids, and nucleic acids, that can be transferred to recipient cells, influencing their functions (Maas et al., 2017). Due to their pivotal role in intercellular communication, EVs have emerged as key players in various cancer hallmarks, such as tumor development, progression, and drug resistance (Kalluri, 2024). Unlike conventional biopsies, EVs provide a dynamic snapshot of genetic and phenotypic heterogeneity within tumors, enabling molecular monitoring of cancer (Santini et al., 2023).

EVs can be categorised based on their size and biogenesis into small (S‐) and large (L‐) EVs. S‐EVs, ranging from 50 to 200 nm, are released either in bulk upon fusion of multivesicular bodies with the plasma membrane (PM) (Verweij et al., 2018) or through single budding events from the PM (ectosomes) (Ai et al., 2024; Pegtel and Gould, 2019). L‐EVs, which include microvesicles (200–1000 nm), apoptotic bodies, and large oncosomes (>1000 nm), are directly released from the PM (Welsh et al., 2024). Recent advances in isolation techniques have enabled molecular characterisation of EV subpopulations and their cargos in several solid tumors (Silva et al., 2025). For example, Vagner and colleagues (Vagner et al., 2018) found an enrichment of tumor DNA signals in L‐EVs (oncosomes) compared to S‐EVs, isolated upon comprehensive differential ultracentrifugation (UC) steps from the plasma of prostate cancer patients. However, this method is generally not considered scalable for clinical practice. Moreover, by using single‐step size exclusion chromatography (SEC) purification of bulk EVs, circulating tumor DNA enrichment within vesicles could not be observed when compared to total plasma (Casanova‐Salas et al., 2024; Moldovan et al., 2024). These discrepancies underscore the importance of a systemic profiling of EV subpopulations to harness their full potential as biomarkers.

The development of EV‐based liquid biopsy tools in hematological malignancies is gaining significant attention, particularly because a substantial proportion of circulating EVs originate from leukocytes that do not necessarily reflect the circulating cell concentration (Holcar et al., 2025).

Diffuse large B‐cell lymphoma (DLBCL) is the most prevalent subtype of non‐Hodgkin lymphoma and exhibits significant biological and clinical heterogeneity. In this context, liquid biopsy may provide a minimally invasive alternative to determine and correlate biological heterogeneity to more personalised therapy. Recent studies (Navarro‐Tableros et al., 2018; Ofori et al., 2021) investigated EV protein composition (Akil et al., 2024; Matthiesen et al., 2022) and the expression of EV‐associated messenger RNAs (EV‐mRNAs) and microRNAs (EV‐miRNAs) in DLBCL (Drees and Pegtel, 2020). Notably, Rutherford et al., 2018. found that EV transcriptomes may, to some extent, mirror the mutations present in malignant B cells (Rutherford et al., 2018).

Ye et al., 2022 leveraged single‐cell RNA sequencing (scRNA‐seq) to construct a comprehensive single‐cell atlas of DLBCL tumors, offering valuable perspectives on the interactions between malignant cells and the tumor microenvironment (TME). Apart from cancer cells, the cellular components of the DLBCL TME, such as macrophages, CD4^+^ T, CD8^+^ T, and NK cells, may provide additional perspectives on how to optimise treatment strategies. Despite significant efforts being made to identify single tumor‐derived EVs in circulation, so far this has not been very successful due to their low abundance and the absence of highly selective tumor markers on the EV surface (Zarovni et al., 2025). We hypothesised that transcriptomic profiling of size‐resolved EV subpopulations, coupled with deconvolution approaches, may better reveal their cellular origins and uncover biologically meaningful tumor‐TME interactions that are masked in bulk EV analyses. Provencio et al. (Provencio et al., 2017) demonstrated the presence of biologically relevant EV‐mRNAs in the plasma of DLBCL patients and reported the prognostic significance of pre‐treatment EV‐associated c‐MYC levels. Additionally, aberrant expression of EV‐miRNAs in DLBCL has been documented, including upregulated miR‐379‐5p, miR‐135a‐3p, and miR‐4476, as well as downregulated miR‐483‐3p and miR‐451a, when compared to healthy donors (HD) (Cao et al., 2022). Other studies have further explored the diagnostic potential of miR‐3960, miR‐6089 and miR‐939‐5p (Caner et al., 2021), as well as the combined diagnostic and prognostic utility of miR‐99a‐5p and miR‐125b‐5p (Feng et al., 2019). These studies highlight the diagnostic potential of EV‐RNAs for DLBCL patients. Nevertheless, to the best of our knowledge, none of these findings have led to diagnostic tests or assays that are used in the clinic.

Most studies using EVs as a liquid biopsy source apply isolation methods that enrich for S‐EVs, ignoring the potential valuable information of L‐EVs. Here, we present the first comprehensive biophysical and molecular (transcriptomic) analysis of both S‐ and L‐EV subpopulations from DLBCL cell lines and patient plasma. Our findings suggest that in patients with DLBCL circulating S‐EVs are predominantly of malignant B cell origin, whereas L‐EVs are derived from cellular components of the TME, revealing previously unappreciated cellular compartmentalisation of EV cargo in DLBCL.

## 2 Materials and Methods

### 2.1 EV Subpopulations and Characterisation

**FIGURE 1:**
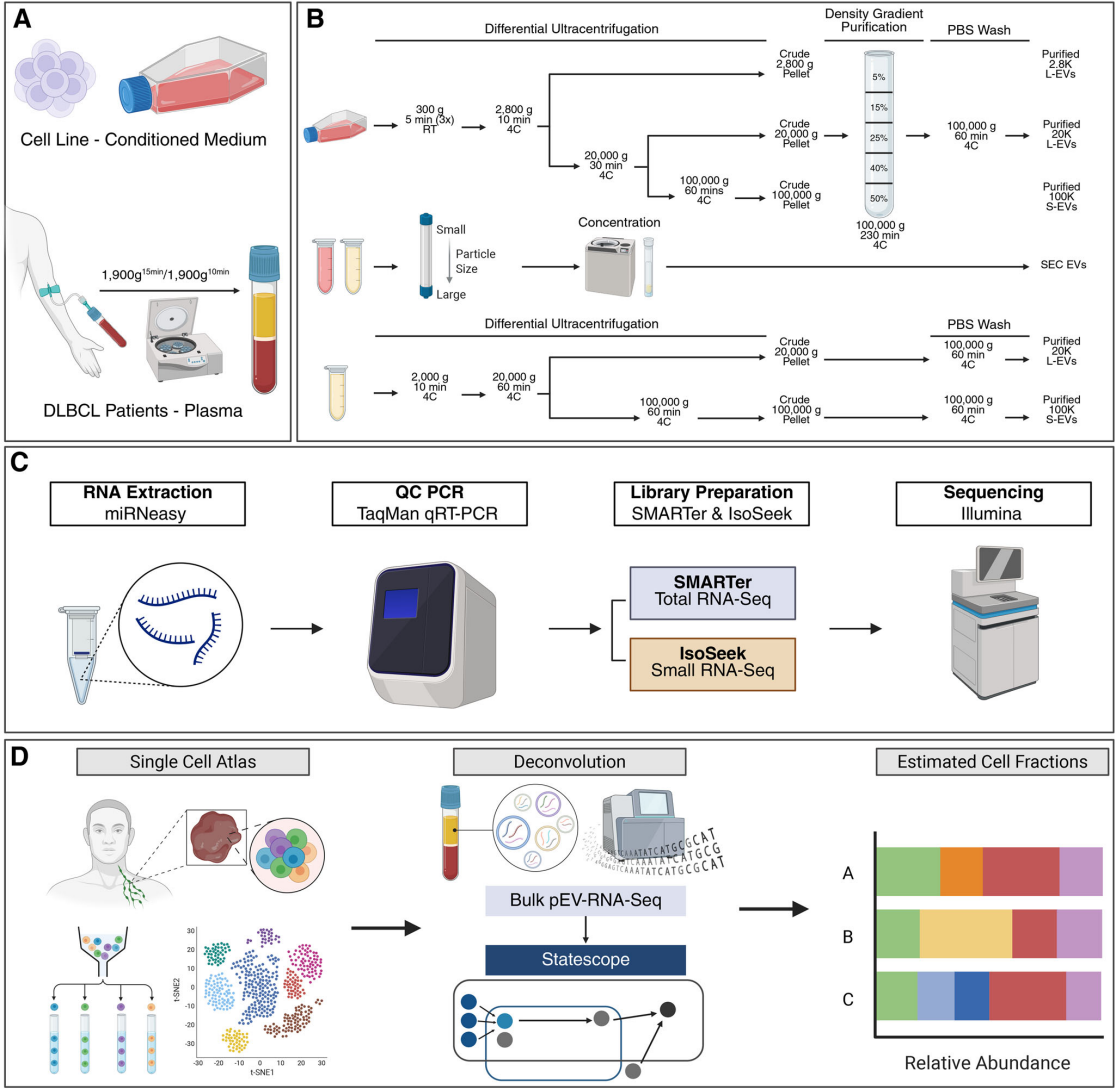
Study design and experimental workflow. (A) EVs sources: conditioned medium from lymphoma cell lines and plasma from patients with DLBCL and HD collected in PAXgene ccfDNA tubes. (B) Workflow for purification EV populations by differential UC followed by DG purification (for cell line‐derived EVs), SEC (for both cell line and plasma EVs) and differential UC (for plasma EVs), yielding very large EVs (UC2.8K VL‐EVs), large EVs (UC20K L‐EVs), small EVs (UC100K S‐EVs), and size‐exclusion chromatography EVs (SEC‐EVs). SEC‐EVs were isolated from 2 mL of concentrated conditioned medium or plasma and then fractions 3 and 4 (500 µL each) were collected, pooled and concentrated. (C) Workflow for EV‐RNA profiling: total RNA sequencing using SMARTer and small RNA sequencing using IsoSeek. (D) Deconvolution of plasma EV‐mRNA with Statescope to estimate cell fractions based on single‐cell RNA‐seq reference profiles.

EV size‐based subpopulations were isolated from conditioned cell culture medium and plasma samples (Figure 1A) using differential UC or automated SEC. Detailed isolation protocols for each method are provided in Figure 1B. Phosphate‐buffered saline (PBS) used to wash and resuspend EV pellets was prefiltered through 0.22 µm filters (Merck, USA). The isolated EVs were characterised using transmission electron microscopy (TEM), tunable resistive pulse sensing (TRPS) analysis, and western blotting (WB) according to the Minimal Information for Studies of Extracellular Vesicles (MISEV) guidelines endorsed by the International Society for Extracellular Vesicles (ISEV) (Welsh et al., 2024). Figures 1C and D, then, illustrate the EV‐RNA profiling and deconvolution workflow.

### 2.2 Cell Lines

All cell lines were cultured following ATCC‐recommended protocols. Specifically, lymphoblastoid RN (passage *x* + 17) and DLBCL SU‐DHL‐4 (passage *x* + 9), U‐2932 (passage *x* + 10), and RI‐1 (passage *x* + 14) cells were maintained in RPMI‐1640 (Gibco, USA) medium supplemented with 10% fetal bovine serum (FBS) (Life Science Production, UK), 2 mM L‐glutamine, and 100 U/ml penicillin/streptomycin (Gibco, USA). Cultures were routinely tested for mycoplasma contamination to ensure authenticity and quality. Cells were cultured at the optimal concentration of 5 × 10^5^ cell/ml. 48 h before the EV isolation, cells were seeded at the concentration of 5 × 10^5^ cells/ml in RPMI‐1640 with 5% EV‐depleted FBS, previously obtained by a 16 h UC at 100,000 g at 4°C and filtration with 0.22 µm filters (Thermo Fisher Scientific, USA).

### 2.3 EV Isolation From Conditioned Cell Medium

*UC*. Conditioned medium (360 mL) was collected and centrifuged three times at 300 g for 5 min at room temperature (RT) to remove cells in suspension and cellular debris. The supernatant was centrifuged at 2,800 *g* for 10 min at 4°C to pellet crude UC2.8K very large (VL‐) EVs. The supernatant was then spun at 20,000 *g* for 30 min at 4°C to pellet crude UC20K L‐EVs and then at 100,000 *g* for 1 h at 4°C to collect crude UC100K S‐EVs. These centrifugation steps were performed using an Optima L‐90K Ultracentrifuge with a SW 32 Ti Swinging‐Bucket Rotor (Beckman Coulter, USA). 2.8K, 20K and 100K pellets were then resuspended in ∼200 µL filtered PBS and subjected to density gradient (DG) purification. Briefly, 50%, 40%, 25%, 15% and 5% solutions were made by diluting a stock solution of iodixanol (Optiprep^TM^, StemCell Technologies, Canada) (diluent: sucrose/NaCl/HEPES pH 7.4 solution). EV samples were mixed in the bottom layer, and then iodixanol solutions were carefully layered at decreasing density to form a discontinuous gradient. The stratification was performed by a 100,000 g UC for 3 h 50 min at 4°C, and EV‐enriched fractions were collected either at 1.10–1.15 g/mL (on top of 25% fraction) for UC2.8K VL‐EVs and UC20K L‐EVs or at 1.10 g/mL (on top of 15% fraction) for UC100K S‐EVs. Purified EVs were then washed in PBS with UC at 100,000 g for 1 h at 4°C, resuspended in filtered PBS, lysis buffer, or Qiazol (Qiagen, USA), depending on downstream analysis, and stored at −80°C until further use. These centrifugation steps were performed using an Optima L‐90K Ultracentrifuge with a SW 40 Ti Swinging‐Bucket Rotor (Beckman Coulter, USA).

*SEC*. The conditioned medium (50 mL) was collected and centrifuged twice at 500 g for 10 min at RT and twice at 2,000 *g* for 15 min at RT. Then aliquots were stored at −80°C until use. For EV isolation, conditioned media were thawed on ice and concentrated using Centricon Plus‐70 filters (Merck Millipore, Ireland). For each sample, we loaded 2 mL (1.5 mL medium + 0.5 mL filtered PBS) onto the automated fraction collector (AFC) (IZON Science, New Zealand) by using qEVoriginal 70 nm columns (IZON Science, New Zealand) and collected fractions 3 and 4 (500 µL each) with a void volume set to the default of 2.7 mL. This procedure was repeated once, yielding a total of four fractions (2 × fraction 3 and 2 × fraction 4). The four fractions were subsequently pooled, resulting in a combined volume of 2 mL SEC‐EVs. EV‐rich fractions were pooled and concentrated by ultrafiltration using 10 kDa Amicon Ultra‐2 mL Centrifugal Filters (Merck Millipore, Ireland) always filled to a total volume of ∼250 µL with filtered PBS and stored at −80°C.

### 2.4 Plasma Samples

We analysed plasma samples from six sex/age‐matched HD enrolled in the Liquid Biopsy Center (Cancer Center Amsterdam) and six DLBCL patients enrolled in the BioLymph study (VUmc METc registration No. 2017.008), an observational biomarker study focusing on lymphoid malignancies. Additional five HD were recruited at IRCCS Azienda Ospedaliero‐Universitaria di Bologna, Institute of Hematology “L. e A. Seràgnoli” (CE AVEC: 995/2021/Sper/AOUBo). The research was conducted in accordance with the Declaration of Helsinki. Supplementary Table 1 provides an overview of the patient and HD characteristics. All participants provided written informed consent. Peripheral blood samples were collected in 10 mL PAXgene Blood ccfDNA tubes (Qiagen, USA) at local centres and shipped to Amsterdam UMC by post. Upon arrival, blood samples were centrifuged for 15 min at 1,900 g at RT. The supernatant was then transferred to a new tube and subjected to a second centrifugation at 1,900 g for 10 min at RT. Platelet‐Free Plasma (PFP) was aliquoted and stored at −80°C until further analysis. All plasma samples had one freeze‐thaw cycle.

### 2.5 Isolation of S‐ and L‐EVs From Blood Samples

*UC*. 2 mL of PFP were thawed on ice and centrifuged at 2,000 g for 10 min at 4°C to remove cell debris. Then, supernatants were centrifuged at 20,000 g for 1 h at 4°C in order to isolate UC20K L‐EVs. The 20K pellet was washed in filtered PBS under the same conditions. The supernatant of L‐EVs was spun at 100,000 g for 1 h at 4°C in order to isolate UC100K S‐EVs. The 100K pellet was then washed in filtered PBS under the same conditions. The resultant EVs were resuspended in filtered PBS, lysis buffer, or Qiazol (Qiagen, USA) depending on downstream analysis, and stored at −80°C until further use. All centrifugation steps were performed using an Optima L‐90K Ultracentrifuge with a SW 40 Ti Swinging‐Bucket Rotor (Beckman Coulter, USA).

*SEC*. 2 mL of PFP were thawed on ice and then fractionated with AFC (IZON Science, New Zealand) by using qEVoriginal 70 nm columns (IZON Science, New Zealand) following the manufacturer's instructions, resulting in SEC‐EVs. The same procedure for EV isolation from cell line conditioned medium was used for plasma samples.

### 2.6 TEM

EV fractions were spotted on freshly glow‐discharged carbon/formvar‐coated mesh grids. EVs sedimented on the grid for 1 min, the excess liquid was blotted off, and the samples were contrasted by 2% uranyl acetate (Cat N. 21447‐25; Polysciences Inc., USA) in water for 1 min. The excess stain was blotted off, and grids were air‐dried. Vesicular structures were imaged with 80 kV Tecnai 12 (Thermo Fisher Scientific, USA) TEM at 60000x magnification using a 2k x 2k pixel CCD side‐mounted camera (Veleta, EMsis GmbH).

### 2.7 TRPS

Concentration and particle size distribution of UC2.8K VL‐EVs, UC20K L‐EVs, UC100K S‐EVs, and SEC‐EVs were analysed by using Exoid (IZON Science, New Zealand). EVs were diluted in filtered PBS and analysed with the NP150, NP800, and NP2000 nm nanopores. IZON calibration particles (CPC800 and CPC2000 for UC2.8K VL‐EVs, UC20K L‐EVs, and SEC‐EVs; CPC200 for UC100K S‐EVs and SEC‐EVs) were then used to calibrate particles’ size and concentration.

### 2.8 WB

WB (Mini‐PROTEAN Tetra system, Bio‐Rad, USA) was performed as per the manufacturer's instructions. Protein lysates were separated using Mini‐PROTEAN TGX Gels (Bio‐Rad, USA) and transferred onto a nitrocellulose membrane. Membranes were then incubated overnight with the following antibodies: mouse anti‐CD63 (1:300; BD Pharmigen^TM^, USA), mouse anti‐CD81 (1:500; BD Pharmigen^TM^, USA), rabbit anti‐Syntenin1 (1:500; abcam, UK), rabbit anti‐HSPA5 (1:500; Cell Signaling, USA), and rabbit anti‐Calnexin (1:1000; MilliporeSigma, USA). The secondary antibodies used were Anti‐rabbit IgG HRP‐linked Antibody (1:1000; Cell Signaling, USA) and Polyclonal Rabbit Anti‐Mouse Immunoglobulin HRP (1:1000; Dako, USA).

### 2.9 Library Preparation and Data Processing

*RNA isolation and quality control polymerase chain reaction (PCR)*. RNA was extracted using the Qiagen miRNeasy Mini kit (Qiagen, USA) according to the manufacturer's instructions. To ensure the RNA quality, we performed a quantitative real time PCR (qRT‐PCR) as previously described (van Eijndhoven et al., 2016) as a quality control step to check the expression of three miRNAs. We profiled EV‐RNA with two library preparation protocols. For total EV‐RNA profiling, we used the SMARTer Stranded Total RNA‐Seq Kit v3 (Takara Bio, USA) according to the manufacturer's instructions, including ribosomal cDNA depletion. A fragmentation step of 3 min at 94°C was used, and the samples were subjected to 5 rounds of amplification in the initial PCR; the final RNA‐seq library amplification PCR consisted of 16 PCR cycles. For EV‐miRNA profiling, small RNA libraries were prepared according to IsoSeek, a unique molecular identifier (UMI)‐informed NGS at single nucleotide resolution (van Eijndhoven et al., 2023). Both libraries were sequenced on the NovaSeq 6000 platform (Illumina, USA) at GenomeScan (Leiden, the Netherlands).

### 2.10 Bioinformatic Processing and Differential Expression (DE) Analysis

#### 2.10.1 SMARTer Stranded Total RNA‐seq

SMARTer Stranded Total RNA sequencing libraries were generated according to the manufacturer's protocol. Adapter trimming was performed using cutadapt (version 4.7) with explicit removal of Illumina TruSeq adapter sequences (Read 1: AGATCGGAAGAGCACACGTCTGAACTCCAGTCA; Read 2: AGATCGGAAGAGCGTCGTGTAGGGAAAGAGTGT) using default quality filtering parameters (minimum length = 20, reads with Q < 20 discarded). Sequencing quality was assessed using FastQC (version 0.12.1) both before and after adapter trimming. UMI processing and deduplication were performed using UMI‐tools (version 1.1.6) with default settings. Filtered reads were aligned to the human reference genome (GRCh38.p14) using STAR (version 2.7.10a). STAR indices were generated from the Ensembl GRCh38.p14 reference genome and RefSeq annotation (NCBI RefSeq) using standard parameters, including sjdbOverhang 100, and alignment was performed using default settings for paired‐end, stranded libraries. Gene‐level read counts were obtained using featureCounts (version 2.0.8) with the following options: paired‐end mode enabled (−p), strandedness specified according to the library preparation (−s 2), assignment at the gene level using Ensembl gene annotations, and exclusion of multi‐mapping reads. Raw gene counts were subsequently normalised to transcripts per million (TPM) using NormSeq (Scheepbouwer et al., 2023). Sequencing quality metrics and alignment statistics for all total RNA‐seq libraries, including read depth, mapping efficiency, and the number of detected genes per EV isolation method, are summarised in Supplementary Table 2.

#### 2.10.2 IsoSeek Small RNA‐seq

Following sequencing with the IsoSeek protocol (van Eijndhoven et al., 2023), the pre‐processing and mapping of adapter‐trimmed reads were performed using the latest version of the sRNAbench command‐line tool (Aparicio‐Puerta et al., 2022). Default parameters were used for all analysis steps after pre‐processing, and MirGeneDB 3.0 (Clarke et al., 2025) was used as the miRNA reference. Quality control of samples was carried out using miRNAQC to rule out technical differences between libraries (Aparicio‐Puerta et al., 2020). Quality control metrics for miRNA‐seq libraries, such as total reads, adapter dimer content, and the number of detected mature miRNAs, are provided in Supplementary Table 3.

#### 2.10.3 Paired‐Design DESeq2 and Robustness Analyses

For paired comparisons of UC20K L‐EVs versus UC100K S‐EVs, DE was performed in DESeq2 using a paired design (∼ patient + condition) and log2 fold‐change shrinkage using apeglm. Robustness was evaluated by leave‐one‐patient‐out (LOPO) sensitivity analysis: each of the 12 paired samples was excluded in turn, DESeq2 was rerun, and for each gene/miRNA the following were calculated: (1) sign consistency of (% LOPO runs matching the full‐data log2FC direction), (2) significance consistency of (% LOPO runs with FDR < 0.05), and (3) effect‐size stability (median, SD, and range of log2FC across LOPO runs). Features were classified as robust if sign consistency ≥ 90% and significance consistency ≥ 80%.

### 2.11 Web‐Based Cell‐Type‐Specific Enrichment Analysis (WebCSEA)

To infer potential cellular origins of differentially expressed transcripts, WebCSEA was performed using the WebCSEA platform available at https://webcsea.mdanderson.org. Gene lists derived from UC20K L‐EVs and UC100K S‐EVs were submitted for enrichment against tissue‐ and cell‐type‐specific signatures. The top 10 general cell‐type categories were ranked by combined log10 *p*‐values and visualised.

### 2.12 EV‐mRNA Deconvolution Using DLBCL scRNA‐seq Reference

*Preprocessing of scRNA‐seq*. Public DLBCL scRNA‐seq was fetched from the CNGB Sequence Archive under accession number CNP0001940. On this dataset, the following quality control was already applied by the original authors: (1) filtering of genes only expressed in less than 3 cells; (2) ambient RNA expression contamination removal by Soup X (Young and Behjati, 2020); (3) doublet removal with Scrublet (Wolock et al., 2019); (4) filtering of cells with less than 500 genes expressed; and (5) filtering of cells with more than 20% of their transcripts coming from mitochondrial genes. After this quality control, raw counts from 24584 genes for 94324 cells were left. Finally, cells from the 3 control samples from 1 Burkitt's lymphoma, 1 follicular lymphoma, and 1 reactive lymphoid hyperplasia were removed, leaving 81,353 cells from 17 samples for the rest of the analyses. Raw counts were then normalised to counts per 10,000 followed by log transformation. From these normalised counts, highly variable genes were selected, which were defined as genes with (1) a mean expression of at least 0.0125 and (2) at most 3 and (3) a minimal dispersion of 0.5. The cell phenotyping from the original authors who published the scRNA‐seq data was used (Ye et al., 2022).

*Creation of scRNA‐seq reference*. From the processed scRNA‐seq, a reference for RNA deconvolution was created. To optimise the genes for discriminative power between the cell types, further gene selection was performed by AutoGeneS (Aliee and Theis, 2021). From these genes the mean gene expression per cell type was extracted, and the gene expression variability was estimated by fitting a LOWESS curve to the mean‐variance trend using the fitTrendVar function from scran (https://s3.jcloud.sjtu.edu.cn/899a892efef34b1b944a19981040f55boss01/bioconductor/3.15/bioc/html/scran.html). Finally, a pseudocount of 0.01 was added to the gene expression variability to circumvent technical problems caused by zero values.

*RNA deconvolution*. Using the scRNA‐seq reference described in the previous section, RNA deconvolution was performed with Statescope (formerly known as OncoBLADE) (Y. Kim et al., 2024) in the following configuration:

$$\alpha_{i}^{t}=1,\kappa_{0j}^{t}=1,\;s=1,\;\alpha_{0j}^{t}=1000$$

and at most 20,000 optimisation iterations, which were repeated 100 times with a different initial guess. Statescope was applied to the bulk RNA‐seq data from (1) small, (2) large, and (3) SEC‐EVs of DLBCL samples or HD separately. The bulk RNA‐seq was preprocessed in the same way as the scRNA‐seq that is normalised to 10,000 counts per sample, log‐transformed and subset on the same genes as selected by AutoGeneS. Leveraging the capacity of Statescope to incorporate the prior expectation of cell type fractions, a near‐zero fraction of 1e‐3 was used as a prior expectation for the malignant cell type for deconvolution of HD. No prior expectations were available for DLBCL samples.

To ensure robustness, Statescope runs were repeated ten times, after which the average estimated fraction over these ten runs was reported.

Validation of DLBCL EV‐mRNA deconvolution was performed through (1) *in silico* spike‐in experiments, where for each S‐ and L‐EV mRNA sample 99%–90% of their bulk EV profile was mixed with 1%–10% of a randomly chosen sample's scRNA‐seq profile of a defined cell type; and (2) simulation experiments using the scRNA‐seq data to assess the recovery of known cell‐type proportions in DLBCL tissue. Deconvolution was repeated ten times to assess reproducibility, and performance was evaluated using Pearson correlation (for relative proportions) and Root Mean Squared Error (RMSE; for absolute proportions).

### 2.13 Statistical Analysis

mRNA‐ and miRNA‐level data were normalised as detailed in section 2.10 using NormSeq (Scheepbouwer et al., 2023) for SMARTer RNA‐Seq (TPM) and RPMlib for IsoSeek miRNA data. For CL‐EV subpopulation comparisons (UC20K L‐EVs versus UC100K S‐EVs), genes/miRNAs were filtered to retain features with ≥ 10 raw counts in at least half of the number of samples of the smaller group (if uneven); this unified filter was applied once and used for all downstream analyses. DE in the main analysis was performed using DESeq2 (version 1.50.2) with a paired design (∼ patient + condition) and apeglm log2 fold‐change shrinkage. Significance criteria were FDR < 0.05 (Benjamini–Hochberg) and |log2FC| > 1 (apeglm‐shrunk). Robustness was assessed by LOPO cross‐validation: each patient's paired samples were iteratively excluded, DESeq2 was rerun, and both unshrunk and shrunk log2 fold changes were computed. Features were classified as robust if sign consistency ≥ 90% and significance consistency ≥ 80% across LOPO runs. Principal component analyses (PCA) used variance‐stabilised (vst) data from DESeq2. Unpaired comparisons used two‐sided Wilcoxon rank‐sum tests. Significance levels: ns (*p* ≥ 0.05), * *p* < 0.05, ** *p* < 0.01, *** *p* < 0.001, **** *p* < 0.0001. Analyses were performed in R (version 4.5.2) with ggplot2 (version 4.0.1).

## 3 Results

### 3.1 EV‐RNA Profiles are Similar Across EV Subpopulations

It is largely unknown whether size‐based EV subpopulations of malignant DLBCL cells can be distinguished based on their RNA cargo. To address this question, we isolated EVs from the conditioned medium of the commonly studied DLBCL cell lines, including SU‐DHL‐4, U2932, and RI‐1, by differential UC followed by DG purification (Silva et al., 2025) in order to enrich different EV populations based on either density (UC2.8K VL‐EVs, UC20K L‐EVs, and UC100K S‐EVs) and/or size by SEC (SEC‐EVs). SU‐DHL‐4 EV subpopulations were characterised as recommended by the MISEV2023 criteria with TEM, TRPS analysis, and WB.

**FIGURE 2:**
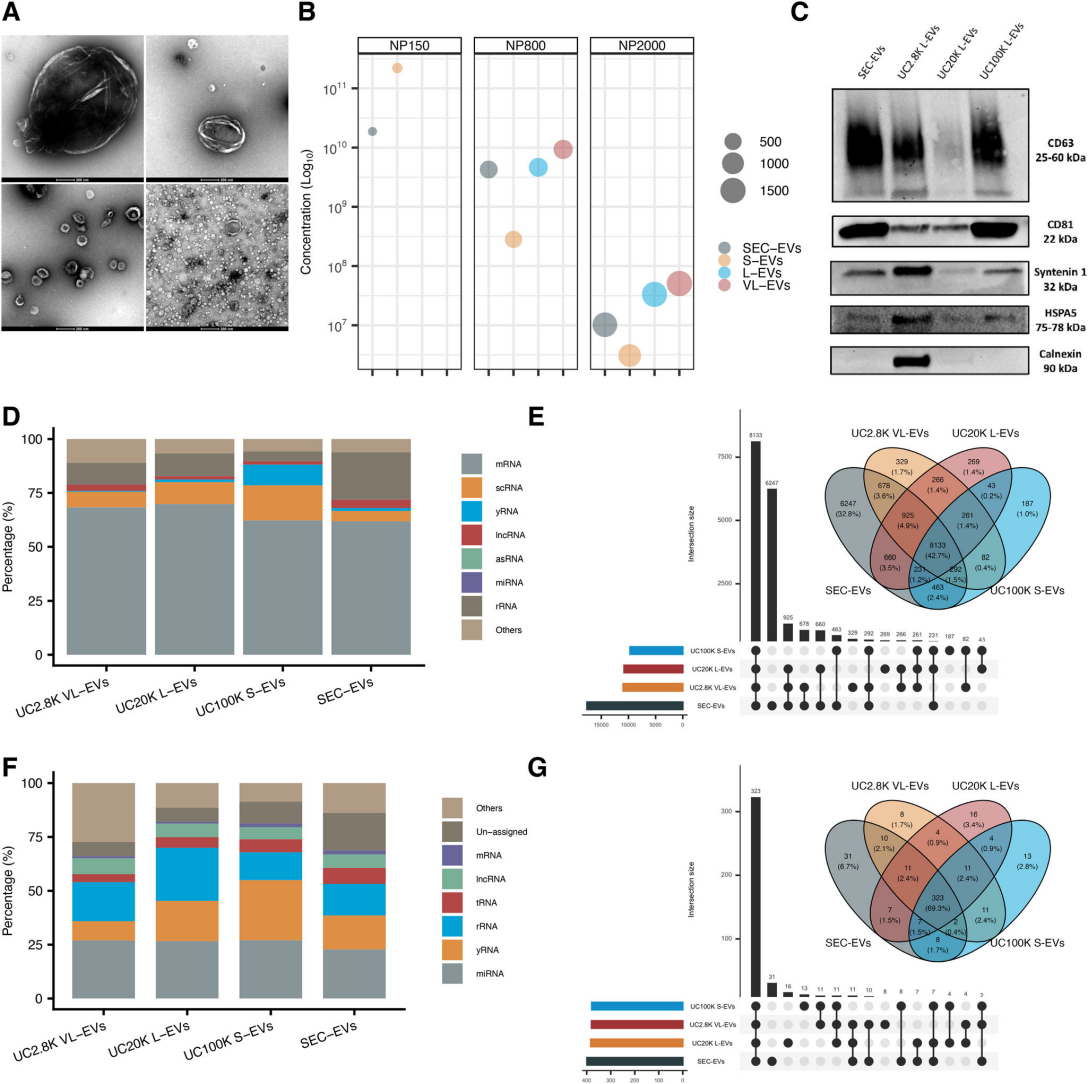
EV subpopulation characterization and EV‐RNA profiling in DLBCL cell lines. (A) TEM analysis of SU‐DHL‐4 EV subpopulations: UC2.8K VL‐EVs (upper left), UC20K L‐EVs (upper right), UC100K S‐EVs (lower left), and SEC‐EVs (lower right). (B) TRPS analysis of SU‐DHL‐4 UC2.8K VL‐EVs, UC20K L‐EVs, UC100K S‐EVs, and SEC‐EVs using EXOID instrument measured by NP150, NP800, and NP2000 nanopores. The measurement of UC2.8K VL‐EVs and UC20K L‐EVs using the NP150 was not possible due to frequent blockage of the nanopore due to the large particles. (C) WB analysis of specific EV markers (CD63, CD81, HSPA5, syntenin 1) and the endoplasmic reticulum marker calnexin in SU‐DHL‐4 EV subpopulations. (D) Distribution of RNA species in total EV‐RNA sequencing using SMARTer protocol. Mean proportions across SU‐DHL‐4, U2932, and RI‐1 cell line EV subpopulations are shown. (E) UpSet plot and Venn diagram showing shared and unique mRNAs across EV subpopulations (UC2.8K VL‐EVs, UC20K L‐EVs, UC100K S‐EVs, SEC‐EVs), using the union of mRNAs detected in SU‐DHL‐4, U2932, and RI‐1 cell lines. (F) Distribution of RNA species in small EV‐RNA sequencing using IsoSeek protocol. Mean proportions across SU‐DHL‐4, U2932, and RI‐1 cell line EV subpopulations are shown. (G) UpSet plot and Venn diagram showing shared and unique miRNAs across EV subpopulations (UC2.8K VL‐EVs, UC20K L‐EVs, UC100K S‐EVs, SEC‐EVs), using the union of miRNAs detected in SU‐DHL‐4, U2932, and RI‐1 cell lines.

UC2.8K VL‐EVs, UC20K L‐EVs, UC100K S‐EVs, and SEC‐EVs were visualised with TEM (Figure 2A), which revealed spherical structures typical of EV morphology. While UC EVs displayed distinct diameter differences, TEM imaging of SEC‐EV samples showed a mixture of particles with varying dimensions, with S‐EVs of < 200 nm dominating the EV‐enriched fractions (Figure 2A). TRPS measurements using three‐sized nanopores identified different EV sizes in all EV subpopulations: UC2.8K VL‐EVs and UC20K L‐EVs exhibited higher concentrations of EVs > 200 nm, while UC100K S‐EVs had the highest concentration of EVs < 200 nm (Figure 2B). TRPS analysis also confirmed the higher concentration of S‐EVs compared to L‐EVs, as previously described (Vagner et al., 2018), and the presence of both S‐ and L‐EVs within SEC‐EVs, although EVs < 200 nm had the highest concentration (Figure 2B). WB detected EV‐specific markers CD63, CD81, Syntenin 1, HSPA5, and calnexin across the EV populations (Figure 2C). In particular, all EV subpopulations were positive for CD63, CD81, Syntenin 1, and HSPA5. Additionally, UC2.8K VL‐EVs expressed the endoplasmic reticulum protein calnexin, as previously described (Crescitelli et al., 2020). These findings confirm the basic morphology and characterisation of four EV subpopulations of various sizes isolated by differential UC and SEC from SU‐DHL‐4 conditioned cell medium.

We next investigated whether EV subpopulations differ in mRNA cargo composition. Using the SMARTer protocol for total EV‐RNA sequencing, we profiled EV‐associated RNAs across four distinct EV subpopulations of SU‐DHL‐4, U2932, and RI‐1. Differences were observed in the mean percentage of mapped mRNA reads: UC2.8K VL‐EVs and UC20K L‐EVs exhibited the highest percentages at 68% and 70%, respectively, while UC100K S‐EVs and SEC‐EVs displayed lower percentages at 62% (Figure 2D). Figure 2E illustrates the overlap of unique EV‐mRNAs among the four subpopulations. Across these subpopulations, we identified a total of 19,066 unique mRNAs (≥ 10 TPM in at least one sample), of which 8,133 (42.7%) were shared among all groups. Correlation analysis revealed that SEC‐EV mRNA profiles correlated strongly with UC20K L‐EVs and UC100K S‐EVs (Pearson correlation, *r* = 0.86 and 0.83, respectively), while SEC‐EVs were least correlated with UC2.8K VL‐EVs (Pearson correlation, *r* = 0.75) (Figure S1A). Overall, EV‐mRNA profiles displayed a relatively high degree of correlation among the four subpopulations, with the lowest correlation observed between UC100K S‐EVs and UC2.8K VL‐EVs (Pearson correlation, *r* = 0.67).

We next applied our IsoSeek small RNA‐seq protocol to analyse the same EV‐size subpopulations of SU‐DHL‐4, U2932, and RI‐1. The mean proportion of RNAs mapped to miRNAs across these three cell lines was 27% in UC2.8K VL‐EVs, UC20K L‐EVs, and UC100K S‐EVs, and 23% for SEC‐EVs (Figure 2F). A total of 466 unique miRNAs (≥ 10 RPM in at least one sample) were detected across these subpopulations, with 323 (69.1%) shared among these size groups (Figure 2G). Pearson's correlation analysis showed a high degree of similarity in EV‐miRNA profiles among the EV‐size subpopulations, with SEC‐EVs correlating with UC2.8K VL‐EVs (Pearson correlation, *r* = 0.95) and UC20K L‐EVs (Pearson correlation, *r* = 0.95), but most strongly with UC100K S‐EVs (Pearson correlation, *r* = 0.98) (Figure S1B). UC100K S‐EVs were least correlated with UC2.8 VL‐EVs (Pearson correlation, *r* = 0.89). These findings indicate notable similarities and some dissimilarities in EV‐miRNA profiles across EV subpopulations and the consistency of miRNA profiles across isolation methods.

### 3.2 Characterisation of Plasma EV Subpopulations and Their EV‐RNA Cargos

**FIGURE 3:**
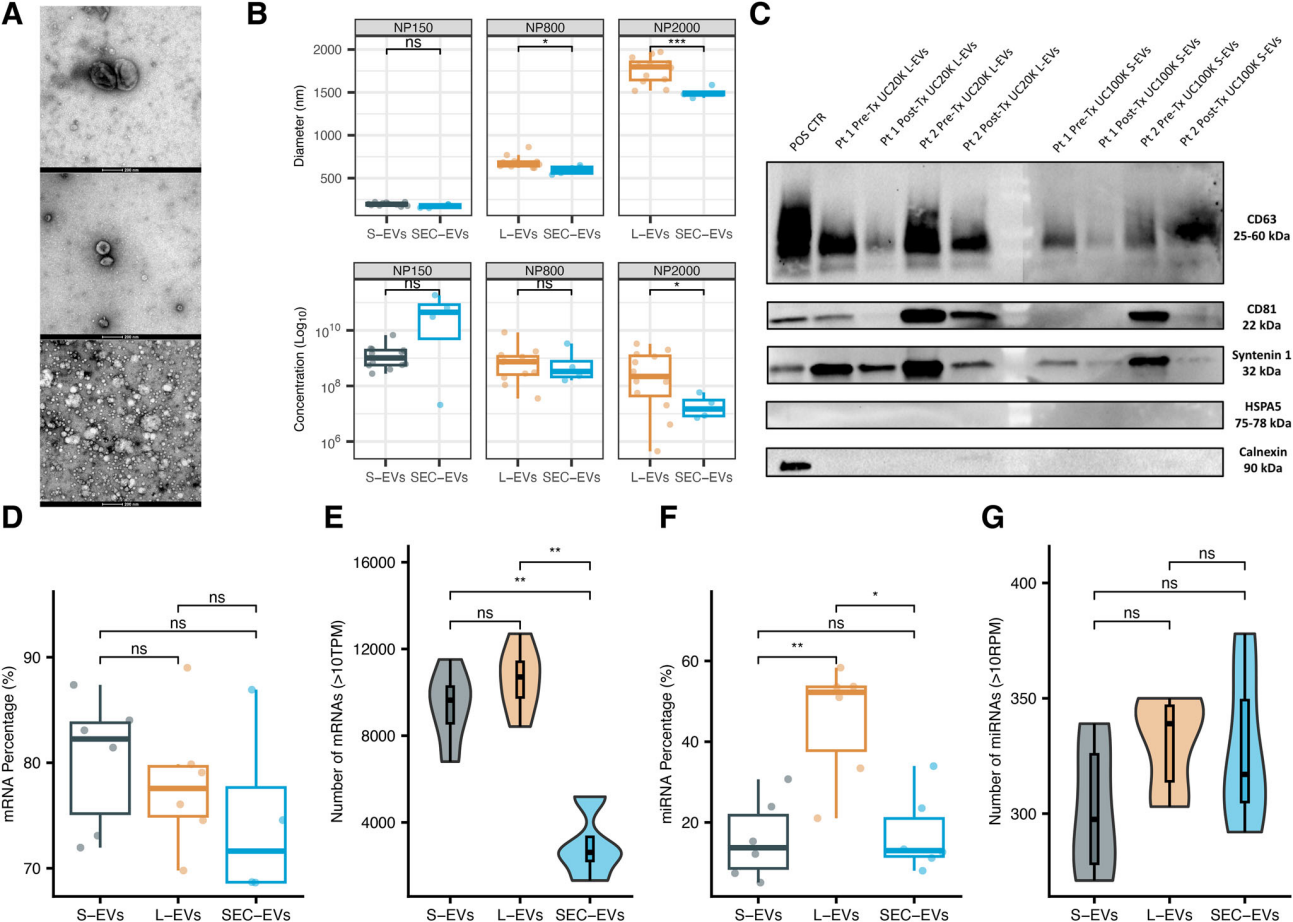
DLBCL plasma EV subpopulation characterization and EV‐RNA profiling. (A) TEM analysis of plasma EV subpopulations: UC20K L‐EVs (upper), UC100K S‐EVs (middle), and SEC‐EVs (lower). (B) TRPS analysis of UC20K L‐EVs, UC100K S‐EVs, and SEC‐EVs using EXOID instrument measured by NP150, NP800, and NP2000 nanopores. Measurements of UC20K L‐EVs using NP150 were excluded due to unreliable results caused by nanopore blockage. (C) WB analysis on specific EV markers (CD63, CD81, HSPA5, syntenin 1) and calnexin in plasma EV subpopulations. Tx indicates treatment timepoint. (D) RNA species distribution from total RNA sequencing using SMARTer protocol across plasma EV subpopulations. (E) Number of normalized mRNA (> 10 TPM) detected across plasma EV subpopulations. (F) miRNA proportion determined by small RNA sequencing using IsoSeek protocol across plasma EV subpopulations. (G) Number of normalized miRNAs (> 10 RPM) detected across plasma EV subpopulations.

For future implementation of EVs as a biomarker source, it is important, if not crucial, to isolate and purify EVs in a standardised and harmonised manner (Drees et al., 2024) and ideally use blood collection tubes that are suitable for delayed processing of the plasma to allow transport from peripheral hospitals and centralised analysis (Dhondt et al., 2023). To this aim, we isolated distinct EV subpopulations from plasma samples of DLBCL patients using differential UC (Bonizzi et al., 2025) and SEC. First, we characterised plasma EV subpopulations, including UC20K L‐EVs, UC100K S‐EVs, and SEC‐EVs, to determine their morphological and size differences. TEM confirmed that all EV populations exhibited spherical morphology (Figure 3A). TRPS measurements provided detailed data on EV diameter and concentration across different nanopore sizes (Figure 3B). Notably, using NP150, the diameters and concentrations of UC100K S‐EVs and SEC‐EVs were similar (216 nm vs. 194 nm, *p* = ns; log10 8.60 vs. 7.32, *p* = ns). However, with NP800, UC20K L‐EVs were significantly larger than SEC‐EVs (681 nm vs. 624 nm, *p* < 0.05), with comparable concentrations (log10 9.05 vs. 8.68, *p* = ns); and, using NP2000, UC20K L‐EVs were substantially larger (1,821 nm vs. 1,586 nm, *p* < 0.001) and had higher concentrations than SEC‐EVs (log10 9.06 vs. 6.94, *p* < 0.05), suggesting that SEC disfavours the isolation of L‐EVs. Moreover, WB further confirmed EV marker presence, revealing higher expressions of CD63 and Syntenin 1 in UC20K L‐EVs compared to UC100K S‐EVs, and a CD81 expression almost restricted to UC20K L‐EVs (Figure 3C). All samples were negative for HSPA5 and calnexin.

The EV‐RNA profiles of the EV subpopulations of DLBCL plasma samples revealed differences. Using the SMARTer protocol, the percentage of mRNAs mapped was similar between UC100K S‐EVs (*n* = 6), UC20K L‐EVs (*n* = 6), and SEC‐EVs (*n* = 4) (80.2%, 78.1%, and 74.7%, *p* = ns) (Figure 3D). UC20K L‐EVs had a similar number of mRNAs > 10 TPM compared to UC100K S‐EVs (*p* = ns), while both UC20K L‐EVs and UC100K S‐EVs had a higher number of mRNAs > 10 TPM than SEC‐EVs (*p* < 0.01 and *p* < 0.01, respectively) (Figure 3E). With the IsoSeek protocol for miRNA profiling, UC100K S‐EVs (*n* = 6) had a similar miRNA mapping percentage compared to SEC‐EVs (*n* = 6) (15.8% vs 17.1%, *p* = ns), while UC20K L‐EVs (*n* = 6) had a higher percentage of small RNAs mapping to miRNAs compared to both SEC‐EVs and UC100K S‐EVs (45.1%, *p* < 0.05 and *p* < 0.01, respectively) (Figure 3F). SEC‐EVs had a similar number of miRNAs > 10 RPM to both UC100K S‐EVs and UC20K L‐EVs (Figure 3G). These findings suggest that the RNA profile across EV subpopulations may differ per RNA species, reflecting the complexity of their RNA cargoes.

### 3.3 Differences in Gene Expression and Cell‐Type‐Specific Enrichment Analysis Between S‐ and L‐EVs From Plasma of DLBCL Patients

**FIGURE 4:**
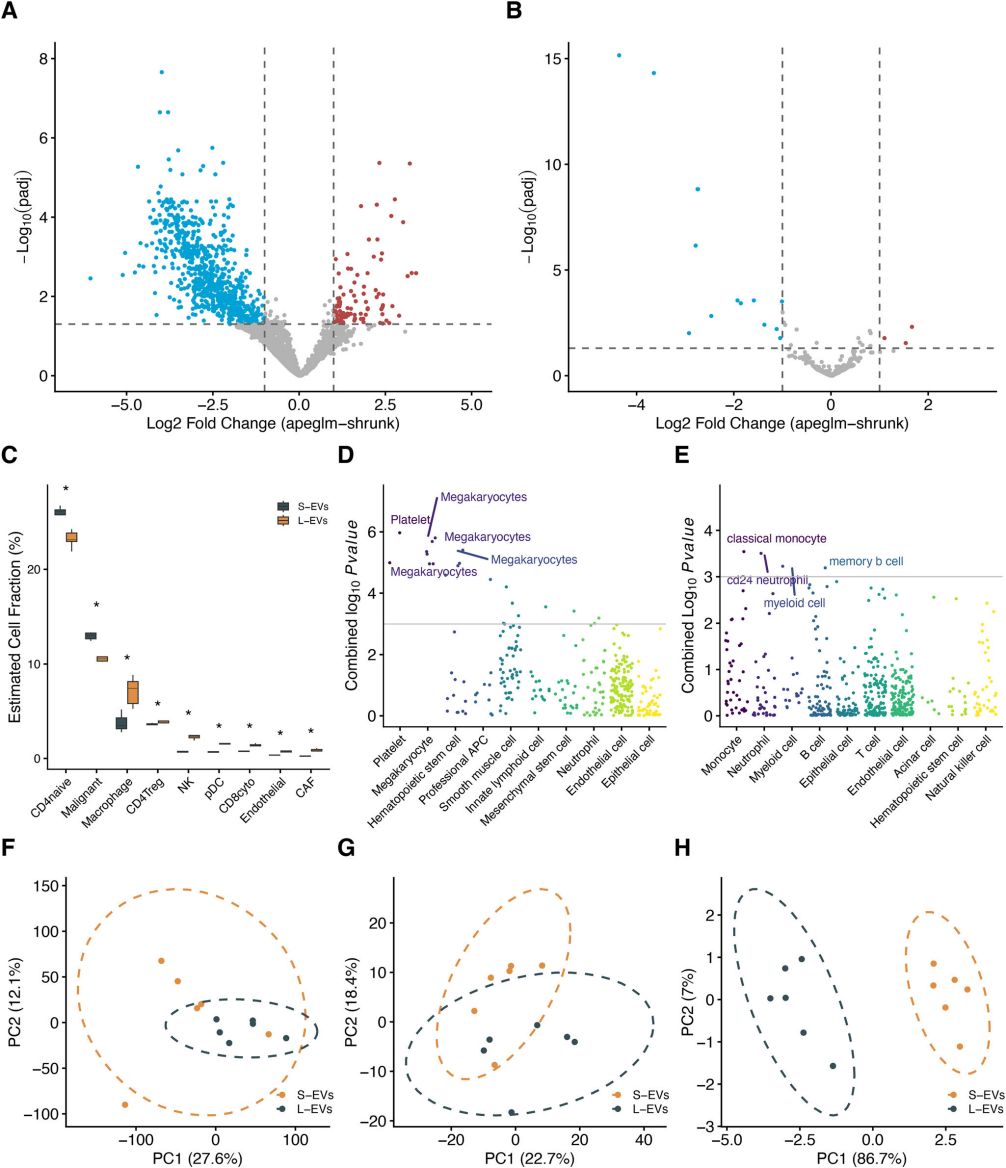
Plasma RNA profiling and deconvolution reveals cell‐type specific EV origins in DLBCL patients. (A) Volcano plot of differentially expressed mRNAs between UC20K L‐EVs (red) and UC100K S‐EVs (blue) in DLBCL patients. Significance level is defined as adjusted *p* value (padj) < 0.05 and |Log2FoldChange (Log2FC)| > 1. (B) Volcano plot of differentially expressed miRNAs between UC20K L‐EVs (red) and UC100K S‐EVs (blue) in DLBCL patients. Significance level is defined as adjusted *p* value (padj) < 0.05 and |Log2FoldChange (Log2FC)| > 1. (C) Estimated cell‐type fraction of UC20K L‐EVs and UC100K S‐EVs through Statescope deconvolution of plasma EV‐mRNA using scRNA‐seq as reference data. (D) Cell‐type enrichment analysis of baseline UC20K L‐EV mRNAs using WebCSEA. (E) Cell‐type enrichment analysis of baseline UC100K S‐EV mRNAs using WebCSEA. (F) PCA of baseline DLBCL plasma UC20K L‐EVs and UC100K S‐EVs based on plasma EV‐mRNA expression profiles. (G) PCA of baseline DLBCL plasma UC20K L‐EVs and UC100K S‐EVs based on plasma EV‐miRNA expression profiles. (H) PCA of baseline DLBCL plasma UC20K L‐EVs and UC100K S‐EVs based on estimated cell fractions by Statescope.

To investigate differences in RNA cargo among EV subpopulations, we compared the RNA profiles of UC100K S‐EVs, UC20K L‐EVs, and SEC‐EVs from DLBCL patients prior to treatment, accounting for paired patient samples. DE analysis identified 99 and 279 enriched genes in UC20K L‐EVs (*n* = 6) and UC100K S‐EVs (*n* = 6), respectively (Figure 4A). In particular, UC100K S‐EVs were enriched in B‐cell tumorigenesis‐related genes, including VAV1 (Hollmann et al., 2010), USP7 (Wu et al., 2022), and WNT2 (Zheng et al., 2023). Conversely, UC20K L‐EVs were enriched in transcripts associated with cell proliferation and apoptosis, such as STK4 (Shang et al., 2022), FLNA (Gachechiladze et al., 2016), NRIP1 (Watanabe et al., 2024), and ERBIN (Supplementary Table 8). PCA of EV‐mRNA expression showed overlap between UC100K S‐EVs and UC20K L‐EVs (Figure 4F). To validate that DE findings were not driven by individual samples, we performed LOPO sensitivity analysis. EV‐mRNA demonstrated strong effect size concordance between the full dataset and LOPO analyses (Pearson *r* ≈ 1.00) (Figure S2A). These findings demonstrate that the observed DE signatures between UC20K L‐EVs and UC100K S‐EVs are robust and not driven by outlier samples. Application of apeglm adaptive shrinkage (Zhu et al., 2019) improved effect size accuracy by reducing noise from low‐count features while preserving biologically meaningful fold changes for highly expressed genes (Figures S2B and C). WebCSEA revealed distinct cellular origins: UC20K L‐EVs (*n* = 6) were associated with megakaryocytes (Figure 4D), while UC100K S‐EVs (*n* = 6) were enriched in genes suggestive of a B‐cell origin (Figure 4E). Then, we compared mRNA profiling of UC20K L‐EVs (*n* = 6) (Figure S4A) and UC100K S‐EVs (*n* = 6) (Figure S4B) with SEC‐EVs (*n* = 4). The comparison yielded DE mRNAs, although PCA displayed modest separation of SEC‐EVs from UC100K S‐EVs and UC20K L‐EVs (Figure S4C). These findings show diverse RNA cargo in EV‐size subpopulations and highlight methods for isolation/purification to reveal these differences. Moreover, the DE analysis of UC20K L‐EVs and UC100K S‐EVs identified a significant number of dysregulated genes between DLBCL patients and HD (Figures S3A and B). PCA analysis (Figure S3C) further demonstrated clear separation by disease status and EV subpopulation, suggesting a potential disease‐related signature in both UC20K L‐EVs and UC100K S‐EVs.

Next, we analysed the EV‐miRNA profiles of the same subpopulations. DE analysis identified a limited number of differentially expressed miRNAs between UC100K S‐EVs (*n* = 6) and UC20K L‐EVs (*n* = 6), with Hsa‐miR154 and Hsa‐miR145 enriched in UC20K L‐EVs and 12 miRNAs, including Hsa‐miR‐10‐5p and Hsa‐miR‐486, enriched in UC100K S‐EVs (Figure 4B) (Supplementary Table 9). EV‐miRNA DE was robust to LOPO analysis (Pearson *r* ≈ 1.00), indicating stability across samples (Figure S2D). apeglm shrinkage reduced low‐count noise while preserving biologically meaningful effect sizes (Figures S2E and F). PCA of baseline EV‐miRNA showed modest separation among subpopulations (Figure 4G). Moreover, compared to SEC‐EVs (*n* = 6), UC20K L‐EVs (Figure S5A) exhibited increased expression of Hsa‐miR‐30 (implicated in ibrutinib resistance (Li et al., 2021)), Hsa‐miR‐21 (Song et al., 2014), Hsa‐miR‐144 (Wang et al., 2016), Hsa‐miR‐340 (promoting tumor‐infiltrating CD8^+^ T cells (Xu et al., 2020)), and Hsa‐miR‐142 (mutated in ∼20% of DLBCL cases (Kwanhian et al., 2012)). In contrast, UC100K S‐EVs (Figure S5B) showed enrichment of Hsa‐miR‐155, a potential prognostic biomarker for therapy response in DLBCL (Zare et al., 2019). Comparison with SEC‐EVs revealed selective enrichment of DLBCL‐related miRNAs, despite a not clear separation through PCA (Figure S5C). Despite these enrichments, the number of differentially expressed miRNAs was limited, consistent with findings in cell line‐derived EVs. In contrast to broad mRNA differences, miRNA differences between EV subtypes were limited in this cohort, suggesting that subtype fractionation may add more value for EV‐mRNA than for bulk EV‐miRNA biomarkers.

### 3.4 EV‐mRNA Deconvolution Reveals Differences in Cell of Origin among EV Subpopulations

To further investigate the cellular origin of EVs by virtue of their transcriptome, we performed deconvolution of EV‐mRNA data using Statescope, leveraging single‐cell sequencing data (Ye et al., 2022) from DLBCL tissues to estimate 15 cell fractions within the TME.

To evaluate the robustness of our approach, we performed an *in silico* simulation using scRNA‐seq data with known ground‐truth cell‐type proportions. We found that EV‐mRNA deconvolution with Statescope reliably recovers relative cell‐type contributions (Figure S6) when consistently increasing spike‐in proportions for memory B cells and CD8^+^ cytotoxic T cells (Figures S6A and B). While absolute fraction estimates exhibited uncertainty, sample ranking by cell‐type proportion was robust. This was further confirmed by deconvolution of pseudo‐bulks from DLBCL tissue scRNA‐seq with Pearson correlation coefficients (PCCs) of > 0.25 across all cell types and all runs (Figure S6C). Additional RMSE analysis indicated a higher absolute error for malignant cell fractions, but relative ordering was preserved with PPCs > 0.75 (Figure S6D). When we averaged cell‐type fractions across repeated deconvolutions, we improved the stability, which was therefore used for further downstream analyses.

Next, we estimated cellular TME fractions using SEC‐EV (*n* = 6) and UC100K S‐EV (*n* = 6) and UC20K L‐EV (*n* = 6) transcriptomic data. Strikingly, UC100K S‐EVs had significantly higher malignant and CD4^+^ naïve T cell fractions compared to UC20K L‐EVs (Figure 4C), while UC20K L‐EVs displayed higher fractions of macrophage, CD4^+^ Treg, NK, and CD8^+^ cytotoxic T cells (Figure 4C and Figure S7A). PCA of deconvoluted cell fractions demonstrated clear separation between UC100K S‐EVs and UC20K L‐EVs, consistent with a different cellular origin of these EV subtypes (Figure 4H). This was much less apparent when using plasma EV‐mRNA and EV‐miRNA profiles alone without deconvolution (Figures 4F and 4G).

SEC‐EVs displayed varying degrees of similarity to both purified UC20K L‐EVs (*n* = 6) and UC100K S‐EVs (*n* = 6), with no significant differences in malignant cell fractions across the three subpopulations (Figure S7A and B). When comparing SEC‐EVs from DLBCL patients (*n* = 4) and HD (*n* = 6), PCA of EV‐mRNA expression profiles showed no clear separation between the two groups despite DE genes (Figure S8A and B). However, after transcriptomic deconvolution, significant differences in fractions of several cell types emerged, resulting in a distinct separation of HD from DLBCL patients (Figure S8C and D). We compared UC20K L‐EVs (*n* = 5) and UC100K S‐EVs (*n* = 5) in five HD and observed a substantial number of differentially expressed genes between these EV subpopulations (Figure S9A). These differences translated into clear separation by PCA (Figure S9B). Likewise, Statescope‐derived cell‐fraction estimates revealed significant shifts across multiple cell types (Figure S9C), and the resulting profiles also segregated cleanly in PCA space (Figure S9D). Overall, these findings suggest that circulating UC20K L‐EVs are more likely derived from non‐tumor cells, potentially immune/stromal cells, while circulating UC100K S‐EVs seem more likely derived from malignant B cells. The distinction between these two may have relevance for future liquid biopsy studies focusing on EV‐RNA.

## 4 Discussion

Most studies that evaluate EVs as an extracellular RNA biomarker source apply methods that enrich S‐EVs (Caner et al., 2021; Cao et al., 2022; Casanova‐Salas et al., 2024; Feng et al., 2019; Jones et al., 2014; Morris et al., 2025; Provencio et al., 2017). Currently, it is unknown whether S‐EVs in circulation of patients with cancer are representative of all cell‐types in the TME. Cell‐type and cell‐state may drive context‐dependent EV‐biogenesis pathways, resulting in packaging of heterogeneous RNA cargo, which may have implications for EV‐based diagnostic assays. Here, we provide a systematic characterisation of EV‐size subpopulations obtained by differential UC and DG enrichment of EV‐size subtypes, adhering to MISEV recommendations (Welsh et al., 2024). We purified UC2.8K VL‐EVs, UC20K L‐EVs, UC100K S‐EVs, and SEC‐EVs from DLBCL cell lines and patient plasma, performed bulk small and messenger RNA sequencing, and determined cellular origins through tumor‐tissue‐informed deconvolution analysis. We demonstrate that different EV‐size populations carry highly similar miRNA cargo. In contrast, mRNA cargo is distinct between EV‐size subtypes and related to a distinct cell‐of‐origin from the TME.

DE analysis showed that UC100K S‐EVs in the plasma of DLBCL patients are enriched in VAV1 transcripts, a gene involved in NFκB activation and CD40‐mediated cell death (Hollmann et al., 2010); USP7, which sustains an active epigenetic program via stabilising MLL2 and WDR5 (Wu et al., 2022); and WNT2, which is overexpressed in DLBCL (Zheng et al., 2023). UC20K L‐EVs, on the other hand, were enriched in transcripts such as TMSB4Y, a potential lymphoma radiotherapy‐resistant gene (Luo et al., 2023); CA2, which is linked to calcium‐dependent signalling in B‐cell lymphomas (Berditchevski et al., 2021); and RAB27B, involved in EV secretion in various cell types (Kunou et al., 2021). Consequently, these signals motivate prospective evaluation of whether EV subtype‐resolved mRNA profiles may be able to track emerging resistance during therapy as suggested by recent data from prostate cancer patients (Casanova‐Salas et al., 2024). Overall, our data reveals molecular heterogeneity of EV subpopulations in patient plasma, suggesting functionally distinct signalling pathways that may contribute to disease progression and immune modulation. Follow‐up studies in a relevant cohort of responders and non‐responders to first‐line therapy will be necessary to determine whether EV‐mRNA profiling can provide actionable information.

In contrast to the broad and biologically coherent mRNA differences, we identified only a limited set of differentially enriched miRNAs between EV subtypes: 3 miRNAs enriched in UC20K L‐EVs and 12 in UC100K S‐EVs. For this reason, enriching for S‐ and L‐EVs is unlikely to increase the sensitivity and/or specificity of total (bulk) EV‐miRNA. In the context of the EV biomarker literature, where miRNAs have often been the primary focus, this relative paucity of robustly changing miRNAs is itself informative. The mRNA and miRNA DE analyses were performed on the same samples, processed in parallel with the same library preparation and DESeq2 pipeline, yet yielded markedly different signal strengths. This pattern argues against low power or sample heterogeneity as the sole explanation for the modest miRNA signal and instead suggests that biologically meaningful differences between S‐ and L‐EVs are more prominent at the mRNA level. Consequently, isolating S‐ and L‐EVs separately is unlikely to substantially improve the sensitivity or specificity of total (bulk) EV‐miRNA biomarkers, whereas DLBCL S‐ and L‐EV mRNA profiling appears more promising.

In addition, in order to delineate the cellular sources of EV subtypes, we developed a deconvolution framework tailored to DLBCL by applying Statescope to EV‐mRNA profiles, using scRNA‐seq reference data from 17 DLBCL tissues (Ye et al., 2022). At diagnosis, PCA of the deconvolved cell‐fraction estimates revealed clear separation between UC100K S‐EVs and UC20K L‐EVs, consistent with distinct cell‐of‐origin contributions to each subpopulation. Variations in cell fractions from which these EVs originate suggest that the substantial differences in DE genes may be attributed to their sources. Specifically, UC20K L‐EVs in circulation of DLBCL patients seem to derive mainly from macrophages, naive CD8^+^ T cells, NK cells, pDCs, and CAFs, while UC100K S‐EVs are secreted by malignant and memory B cells. This distinct cellular origin of UC20K L‐EVs and UC100K S‐EVs suggests functional specialisation of EV subpopulations in DLBCL biology, as previously suggested in other tumor contexts (Muhsin‐Sharafaldine et al., 2016; Patton et al., 2020; Tamborini et al., 2025). So, our hypothesis is that L‐EVs, primarily deriving from immune/stromal cells, may mediate TME‐driven functions that support lymphoma niche, while S‐EVs, mainly secreted by malignant and memory B cells, may propagate tumor cell‐specific oncogenic programs. Overall, this strategy appears useful for the interrogation of the TME through EV analysis, highlighting the potential of EV transcriptomics as a liquid biopsy method for cancer patients (Shi et al., 2020). Future studies should investigate the correlation between plasma EV‐derived TME signatures and clinical outcomes to assess their predictive and prognostic utility.

At the same time, the deconvolution results should be interpreted with caution. Statescope was originally optimised and validated on bulk tissue transcriptomes (Y. Kim et al., 2024), not with EV‐derived RNA. EV‐mRNA profiles may be subject to bias in cargo selection, packaging, and release. Emerging evidence suggests that several tissue‐based deconvolution frameworks can be applied to EV‐RNA datasets with reasonable accuracy (Jensen et al., 2025), but systematic benchmarking is required for future clinical implementation. Further work incorporating EV‐specific reference datasets, orthogonal experimental positive and negative controls, and, ideally, single‐cell or single‐particle‐resolved EV approaches may improve robustness and validate deconvolution tools in a diagnostic context. In addition, while we evaluated multiple DLBCL cell lines, cell‐to‐cell heterogeneity persists even within established cell lines, and bulk profiling of S‐ and L‐EVs may obscure more granular differences. Future studies employing single‐cell and single‐vesicle approaches could provide higher‐resolution insight into how cellular variability influences EV composition.

Our results further suggest that RNA cargo composition is strongly influenced by the EV isolation method. Several DLBCL‐related transcripts (e.g., ROR1) and miRNAs (including hsa‐miR‐21, hsa‐miR‐142, and hsa‐miR‐155) were relatively enriched in UC‐derived EVs compared with SEC‐EVs, highlighting that biologically relevant signals can be modulated—or even masked—by pre‐analytical choices. This has direct implications for the comparability of EV studies and for the design of translational biomarker pipelines.

For the translational utility of EV‐RNAs as biomarkers, SEC offers several practical advantages over differential UC, including reduced labour intensity, shorter processing time, and enhanced standardisation (as established by the IZON Quality Management System), as we recently showed for patients with Hodgkin lymphoma (Drees et al., 2021). In addition, although SEC is designed to enrich S‐EVs, our data indicate that it recovers a mixture of S‐ and L‐EVs, which may be advantageous when the aim is to capture a broad EV repertoire. Differential UC, on the other side, can specifically enrich for specific EV subtypes and may yield important biological insights. Since, according to our results, the RNA cargo differs between S‐ and L‐EVs, this technique could be useful to study the different functional effects of enriched EV subtypes on receiving cells in the context of DLBCL. However, because differential UC is not scalable, new methods are urgently needed. Recently proposed high‐throughput and automated approaches (Bergqvist et al., 2025) and immuno‐isolation strategies, such as surface epitope immunoaffinity isolation (Khanabdali et al., 2024), offer a route toward reducing labor‐intensive and non‐scalable procedures and shortening processing time while maintaining specificity. To fully realise this potential, firstly, robust surface markers that uniquely distinguish L‐EVs from S‐EVs must be identified in patient plasma EVs.

Our data further underscore that plasma‐derived EVs are highly sensitive to pre‐analytical factors, including collection tube type, centrifugation protocols, and platelet contamination (Bracht et al., 2023; Dhondt et al., 2023; Rikkert et al., 2018). We deliberately used a dual‐spin plasma preparation akin to the ISTH‐endorsed protocol (Lacroix et al., 2012) to minimise platelet contamination and cellular debris and to enrich small and medium‐sized EVs. A consequence of this choice is that VL‐EVs (>1000 nm) are largely excluded from the final preparation. Although VL‐EVs may harbour distinct cargos and functions, our aim in the present study was to comprehensively profile the predominant circulating EV subpopulations within the analytical range of our isolation workflow. Thus, the absence of VL‐EVs represents a clear limitation but does not undermine the robustness of the observations regarding S‐ and L‐EVs. A more complete understanding of the circulating EV landscape will require future studies that explicitly include VL‐EVs, for example, by employing complementary techniques such as asymmetric flow field‐flow fractionation (AF4), which can separate and characterise larger EVs and lipoprotein‐sized particles (Y. B. Kim et al., 2022; Zhang et al., 2018).

This study has additional limitations. First, although SEC‐EVs represent a mix of both S‐ and L‐EVs, TRPS analysis showed that they do not comprise the fraction of larger EVs (> 1500 nm) found within the UC20K L‐EV pellet. Moreover, despite its high accuracy, the measurement of particle size and concentration with TRPS is influenced by the nanopore size that is applied. This was particularly evident for our attempts to measure UC2.8K VL‐EVs and UC20K L‐EVs using the NP150 (150 nm pore size) on the Exoid apparatus (IZON, New Zealand). Indeed, the concentration estimations become unreliable due to frequent blockage of the nanopore due to the large particles. Second, deconvolution in this study was performed using a tissue scRNA‐seq reference and a tissue‐validated deconvolution method. A recent review of EV deconvolution techniques has shown that other tissue‐validated deconvolution methods perform well in EV‐mRNA deconvolution (Jensen et al., 2025), suggesting transferability between sample types. However, experimental validation of the deconvolution‐based findings in this work is still required. Our analyses of EV‐RNA cargos, deconvolved TME contributions, and EV isolation method‐dependent signals should therefore be viewed as mechanistic and hypothesis‐generating. Larger, prospectively collected cohorts with standardised pre‐analytics and longitudinal sampling will be needed to determine whether discriminating between S‐ and L‐EVs adds clinically actionable information beyond bulk EV profiling or other circulating biomarkers. Moreover, future studies integrating functional assays of S‐ and L‐EVs in recipient cells will be important to connect EV molecular cargo with downstream biological effects and to extend the findings of the present transcriptomic and deconvolution analyses.

In conclusion, this study provides the first comprehensive profiling of EV‐RNA cargos in size‐defined EV subpopulations derived from DLBCL cell lines, DLBCL patient plasma, and HD plasma. Our data demonstrate that EV size is associated with distinct transcriptomic signatures and inferred cellular origins, with S‐ and L‐EVs capturing complementary aspects of the DLBCL TME. While miRNA differences between EV subpopulations are relatively modest, mRNA cargos reveal richer, functionally coherent patterns related to signalling, apoptosis, and immune interactions. These findings support the concept that EV transcriptomics, particularly when preserving EV size heterogeneity and integrating deconvolution, can serve as a powerful liquid biopsy strategy to interrogate tumor and microenvironmental biology in DLBCL. Future studies should test whether these EV‐derived signatures can be leveraged for response prediction, minimal residual disease monitoring, and rational selection of targeted therapies.

## Author Contributions

**Filippo Maltoni**: conceptualization, methodology, investigation, formal analysis, writing – original draft, writing – review and editing. **Steven Wang**: conceptualization, methodology, investigation, formal analysis, writing – original draft, writing – review and editing. **Mischa F.B. Steketee**: investigation, formal analysis, writing – review and editing. **Cristina A. Gómez‐Martín**: investigation, formal analysis, writing – review and editing. **Esther E.E. Drees**: sample acquisition, writing – review and editing. **Federica Morelli**: investigation, writing – review and editing. **Leontien Bosch**: investigation, writing – review and editing. **Monique van Eijndhoven**: investigation, writing – review and editing. **Gert Jan Timmers**: sample acquisition, writing – review and editing. **Ilse Houtenbos**: sample acquisition, writing – review and editing. **Josée M. Zijlstra**: sample acquisition, writing – review and editing. **Xiaofei Ye**: generation of reference data. **Qiang Pan‐Hammarström**: generation of reference data. **Martine E.D. Chamuleau**: sample acquisition, writing – review and editing. **Pier Luigi Zinzani**: sample acquisition, writing – review and editing. **Yongsoo Kim**: methodology, writing – review and editing, supervision. **Lucia Catani**: conceptualization, methodology, writing – review and editing, supervision. **D. Michiel Pegtel**: conceptualization, methodology, funding acquisition, writing – review and editing, supervision.

## Funding

This research received no external funding.

## Ethics Approval and Consent Statement

The BioLymph study was approved by the Institutional Review Board (VUmc METc registration No. 2017.008) and conducted in accordance with the Declaration of Helsinki. The research was approved by the institutional review board of Area Vasta Emilia Centro (AVEC) Ethical Committee (CE AVEC: 995/2021/Sper/AOUBo) and conducted in accordance with the Declaration of Helsinki. All participants provided written informed consent prior to participation.

## Conflicts of Interest

Michiel Pegtel was the CSO of ExoBiome B.V., received grant support from Takeda, Amgen, Abbvie and Gilead not related to this project. The other authors declare that they have no competing interests.

## Supporting information




**Supporting Information**: jev270259‐sup‐001‐Tables.xlsx

**Supporting Information**: jev270259‐sup‐002‐SuppMat.pdf

## Data Availability

Sequencing data generated have been deposited in the Sequence Read Archive (SRA) of the National Center for Biotechnology. Information under accession number PRJNA1231995. Data requests will be reviewed by the Data Access Committee (DAC) to determine whether the request meets the local and European regulations on privacy. Please contact D. M. Pegtel for data requests. Sequencing data will be accessible on https://dataview.ncbi.nlm.nih.gov/object/PRJNA1231995?reviewer=tskjt1lv97mrfk7pqimfoeg7dv. We have submitted all relevant data of our experiments to the EV‐TRACK knowledgebase (EV‐TRACK ID: EV250130) (Van Deun et al., 2017).
