# Supplementary material for: Small and Large Extracellular Vesicles in Circulation of Diffuse Large B‐Cell Lymphoma Patients Originate From Different Cell Types of the Tumor Microenvironment
Source: J Extracell Vesicles. 2026 Mar 26;15(4):e70259. doi: 10.1002/jev2.70259 (PMC13140527; doi:10.1002/jev2.70259)
Supplement: Supplementary file 2 — Supporting Information: jev270259‐sup‐002‐SuppMat.pdf [file JEV2-15-e70259-s002.pdf]

Supplementary Information

Small and Large Extracellular Vesicles in Circulation of Diffuse Large B-Cell Lymphoma Patients Originate from Different Cell Types of the Tumor Microenvironment

Filippo Maltoni[1,2]*, Steven Wang[3,5]*, Mischa. F.B. Steketee[4], Cristina A. Gómez-Martín[4,5], Esther E.E. Drees[4,5], Federica Morelli[1], Leontien Bosch[4], Monique van Eijndhoven[4], Gert Jan Timmers[6], Ilse Houtenbos[7], Josée M. Zijlstra[3], Xiaofei Ye[8], Qiang Pan-Hammarström[9], Martine E.D. Chamuleau[3,5], Pier Luigi Zinzani[1,2], Yongsoo Kim[4#], Lucia Catani[1,2#], D. Michiel Pegtel[4,5#]

*Filippo Maltoni and Steven Wang contributed equally to this work (co-first authors).
#Yongsoo Kim, Lucia Catani, D. Michiel Pegtel contributed equally to this work (co-senior authors).

1. Department of Surgical and Medical Sciences, Institute of Hematology "L. e A. Seràgnoli", University of Bologna, Bologna, Italy
2. IRCCS Azienda Ospedaliero-Universitaria di Bologna, Institute of Hematology "L. e A. Seràgnoli", Bologna, Italy
3. Amsterdam UMC Location Vrije Universiteit Amsterdam, Hematology, De Boelelaan 1117, Amsterdam, The Netherlands
4. Amsterdam UMC Location Vrije Universiteit Amsterdam, Pathology, De Boelelaan 1117, Amsterdam, The Netherlands
5. Cancer Center Amsterdam, Imaging and Biomarkers, Amsterdam, The Netherlands
6. Amstelland Ziekenhuis, Amstelveen, The Netherlands
7. Spaarne Gasthuis, Haarlem, The Netherlands
8. Department of Biosciences and Nutrition, Karolinska Institutet, Stockholm, Sweden
9. Division of Immunology, Department of Medical Biochemistry and Biophysics, Karolinska Institutet, Stockholm, Sweden

Correspondence
D. Michiel Pegtel, Amsterdam UMC Location Vrije Universiteit Amsterdam, Pathology, De Boelelaan 1117, Amsterdam, The Netherlands
E-mail: d.pegtel@amsterdamumc.nl

Supplemental Information

Supplementary Figures

Supplementary Figure 1. Pearson Correlation Analysis of EV-RNA and EV-miRNA Profiles across EV Subpopulations of DLBCL Cell Line Supernatant.

Supplementary Figure 2. Robustness and Quality Control of Differential Expression Analysis.

Supplementary Figure 3. Differentially Expressed EV-mRNAs in UC20K L-EVs and UC100K S-EVs from HD and DLBCL patients.

Supplementary Figure 4. Differentially Expressed EV-mRNAs in SEC-EVs compared with UC20K L-EVs and UC100K S-EVs from Baseline DLBCL patients.

Supplementary Figure 5. Differentially Expressed EV-miRNAs in SEC-EVs compared with UC20K L-EVs and UC100K S-EVs from Baseline DLBCL patients.

Supplementary Figure 6. Simulation-based validation of EV-mRNA deconvolution performance.

Supplementary Figure 7. Cell-Type Deconvolution of EV subpopulations from Paired Baseline DLBCL Patients.

Supplementary Figure 8. SEC-EV mRNA Signatures and Inferred Cell-Type Composition in HD and DLBCL Patients.

Supplementary Figure 9. Differential Expression and Deconvolution of EV Subpopulations in HD.

Supplementary Tables*

Supplementary Table 1. Patient and Healthy Donor Characteristics.

Supplementary Table 2. SMARTer Mapped EV-RNA Species.

Supplementary Table 3. IsoSeek Mapped EV-RNA Species.

Supplementary Table 4. Quality Control of SMARTer.

Supplementary Table 5. Quality Control of IsoSeek.

Supplementary Table 6. SMARTer EV-mRNA Read Counts.

Supplementary Table 7. IsoSeek EV-miRNA Read Counts.

Supplementary Table 8. Differentially Expressed EV-mRNAs between UC20K L-EVs and UC100K S-EVs (Reference) in Baseline DLBCL Patients.

Supplementary Table 9. Differentially expressed miRNAs between UC20K L-EVs and UC100K S-EVs (Reference) in baseline DLBCL patients.

Supplementary Table 10. Average Cell Fraction Estimated by Statescope.

*Supplementary Tables can be found in the separate Supplementary Tables file. This document contains only legends of supplementary tables.

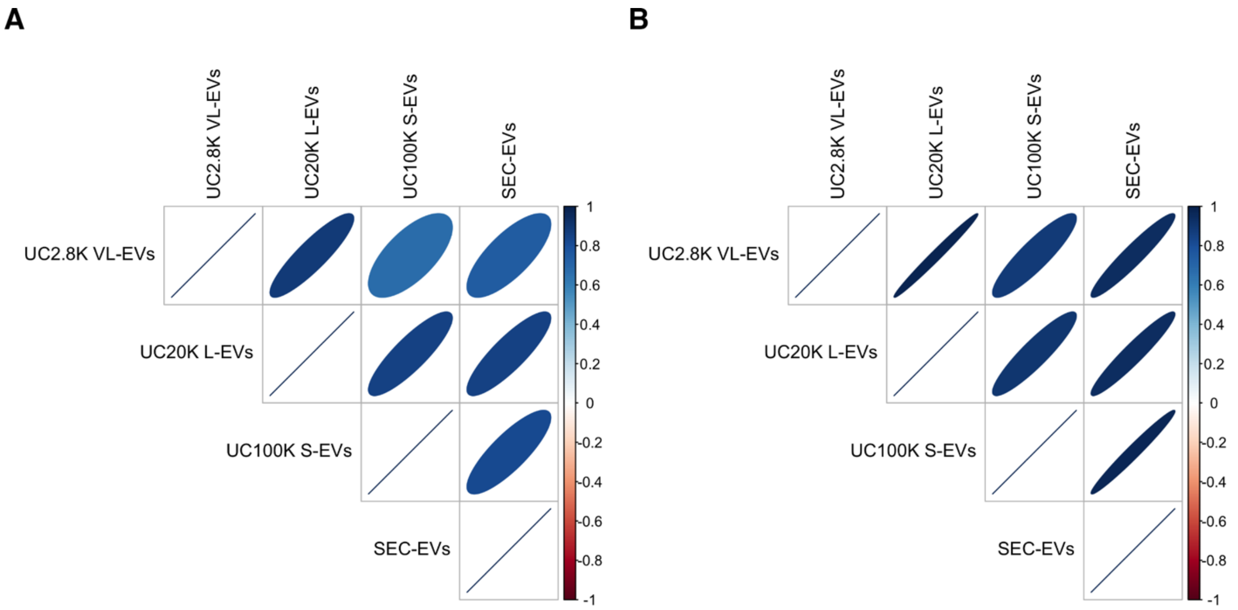

Supplementary Figure 1. Pearson Correlation Analysis (PCA) of EV-RNA and EV-miRNA Profiles across EV Subpopulations of DLBCL Cell Line Supernatant.

(**A**) Correlation of EV-mRNA expression profiles (> 10 TPM) among UC2.8K VL-EVs, UC20K L-EVs, and UC100K S-EVs, and SEC-EVs derived from SU-DHL-4, U2932, and RI-1 cell line supernatant. (**B**) Correlation of EV-miRNA expression profiles (> 10 RPMlib) among corresponding EV subpopulations from the same cell lines. Abbreviations: TPM, transcripts per million; RPMlib, reads per million mapped to the miRNA library size.

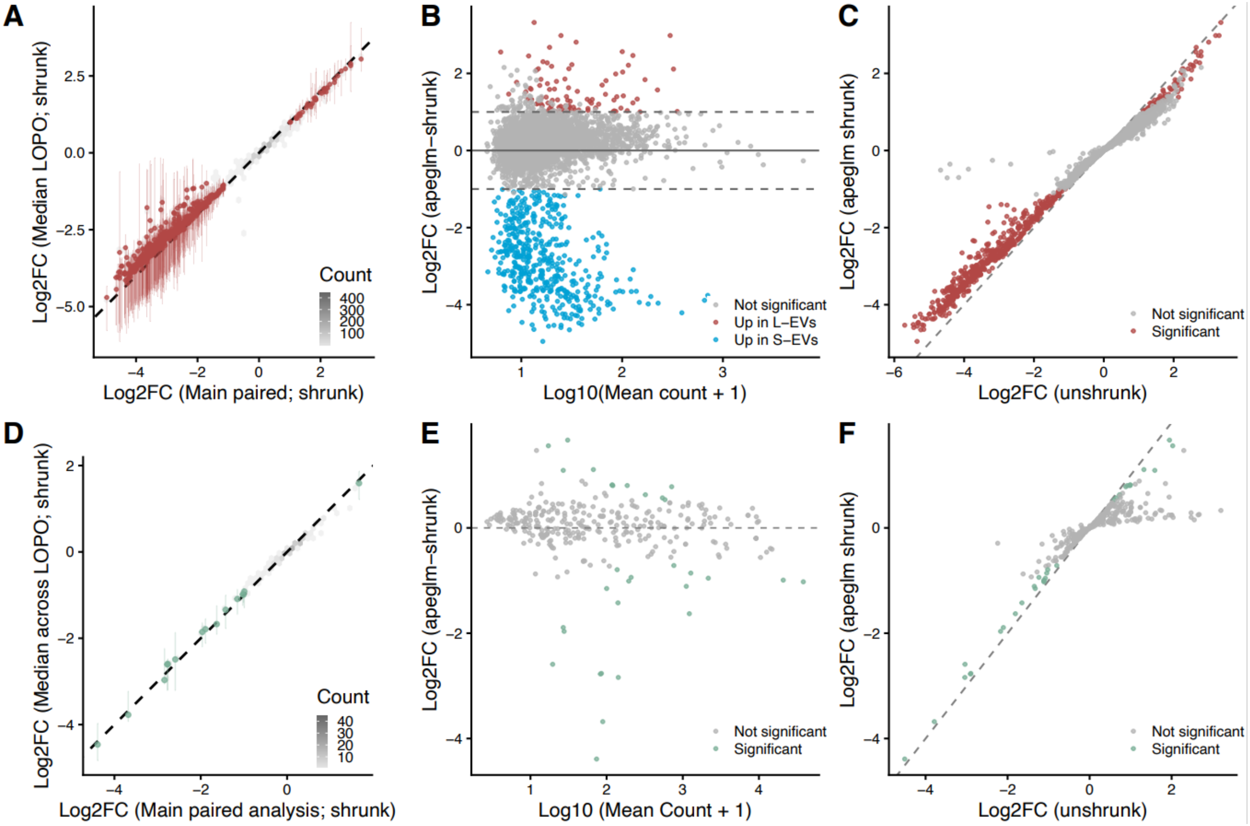

Supplementary Figure 2. Robustness and Quality Control of Differential Expression Analysis. (**A, D**) Leave-one-patient-out (LOPO) cross-validation demonstrates robustness of differential expression results. Hexagonal bins (grayscale gradient) show mRNA/miRNA density. Each point represents the log2 fold change (L-EVs vs S-EVs; apeglm-shrunk) from the main paired analysis (x-axis) versus the median across LOPO iterations (y-axis). Red (mRNA) or green (miRNA) points highlight robust differentially expressed features (padj < 0.05, sign consistency $\geq 90\%$, significance consistency $\geq 80\%$). Error bars show the min-max range across LOPO runs. Dashed diagonal line represents perfect concordance ($y = x$). Strong correlation indicates results are not driven by individual patients. (**B, E**) MA plots show the relationship between mean expression level (x-axis) and apeglm-shrunk log2 fold change (y-axis). For mRNA (B): red = up in L-EVs, blue = up in S-EVs, gray = not significant. For miRNA (E): green = significant (padj < 0.05), gray = not significant. Horizontal dashed lines mark $|\log2FC| = 1$ threshold. (**C, F**) Shrinkage scatter plots compare unshrunk (Wald test; x-axis) versus apeglm-shrunk (y-axis) log2 fold change estimates. Red (mRNA) or green (miRNA) = significant DEGs/DEMs (padj < 0.05); gray = not significant. Dashed diagonal = no shrinkage ($y = x$). Apeglm moderates extreme fold change estimates for features with high uncertainty while preserving well-supported estimates.

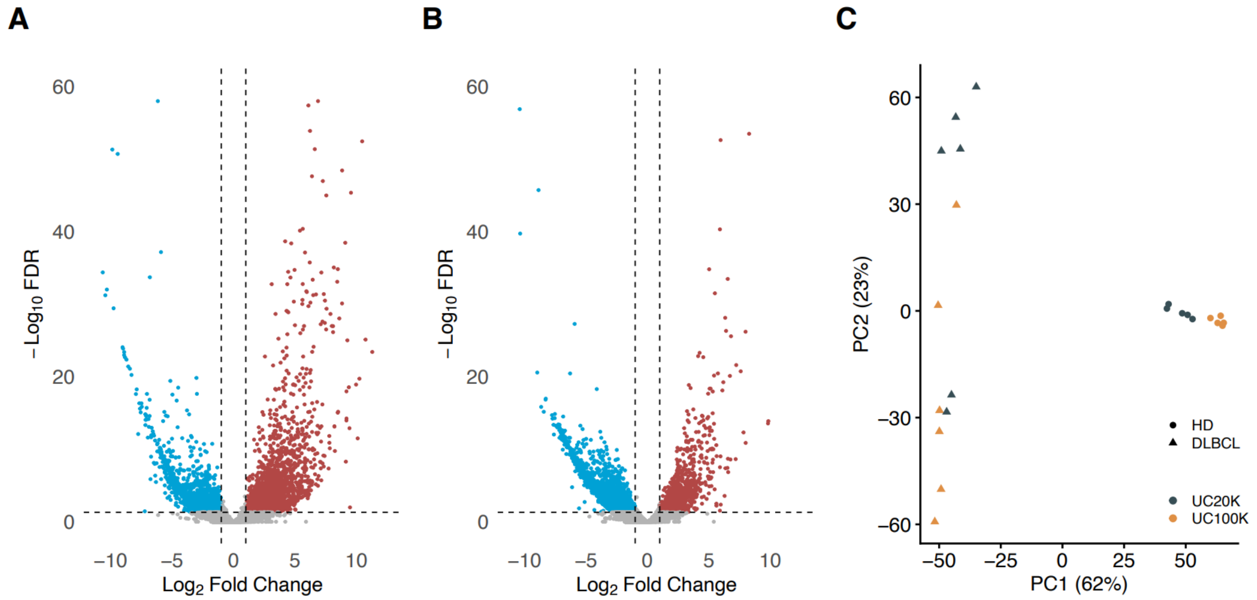

Supplementary Figure 3. Differentially Expressed EV-mRNAs in UC20K L-EVs and UC100K S-EVs from HD and DLBCL patients.

(**A**) Volcano plot of differential mRNA expression in UC20K L-EVs comparing DLBCL patients to HD. (**B**) Volcano plot of differential mRNA expression in UC100K S-EVs comparing DLBCL patients to HD. (**C**) PCA of UC20K L-EV and UC100K S-EV mRNA expression profiles from HD and DLBCL patients, colored by EV subpopulations and shaped by diagnosis. Significantly deregulated transcripts are defined as an absolute log2 fold change > 1 and a Benjamini-Hochberg-adjusted p value (FDR) < 0.05.

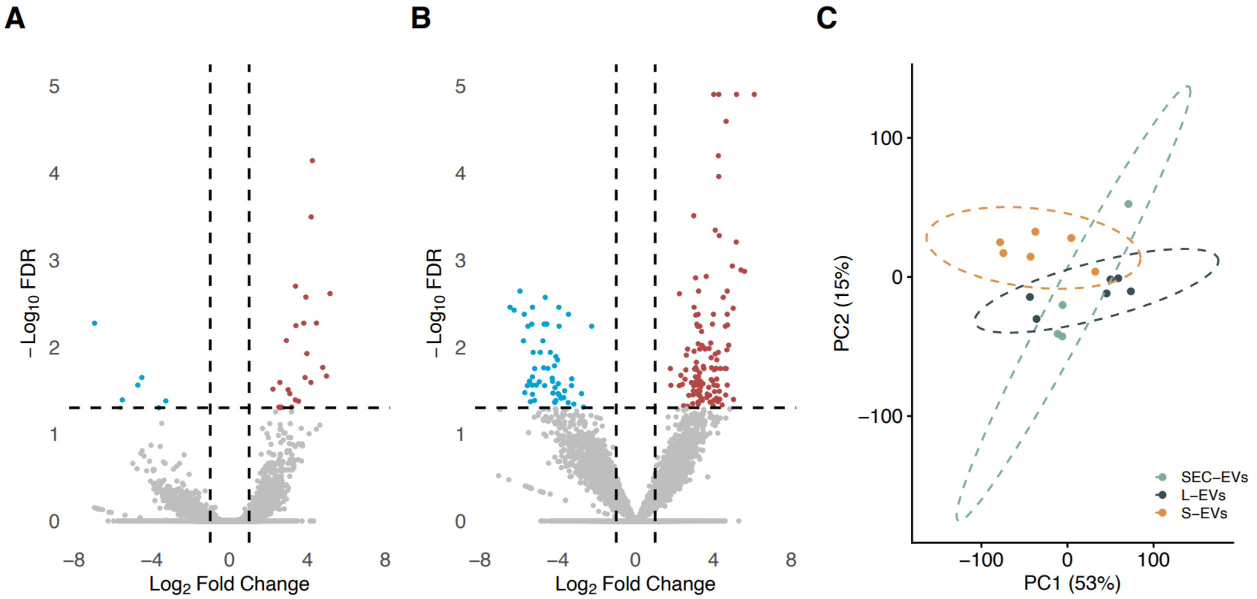

Supplementary Figure 4. Differentially Expressed EV-mRNAs in SEC-EVs compared with UC20K L-EVs and UC100K S-EVs from Baseline DLBCL patients.

(**A**) Volcano plot of differential EV-mRNA expression in SEC-EVs versus UC20K L-EVs. (**B**) Volcano plot of differential EV-mRNA expression in SEC-EVs versus UC100K S-EVs. (**C**) PCA of EV-mRNA expression profiles from SEC-EVs, UC20K L-EVs, and UC100K S-EVs from DLBCL patients. Significantly deregulated transcripts are defined as an absolute log2 fold change > 1 and a Benjamini-Hochberg-adjusted p value (FDR) < 0.05.

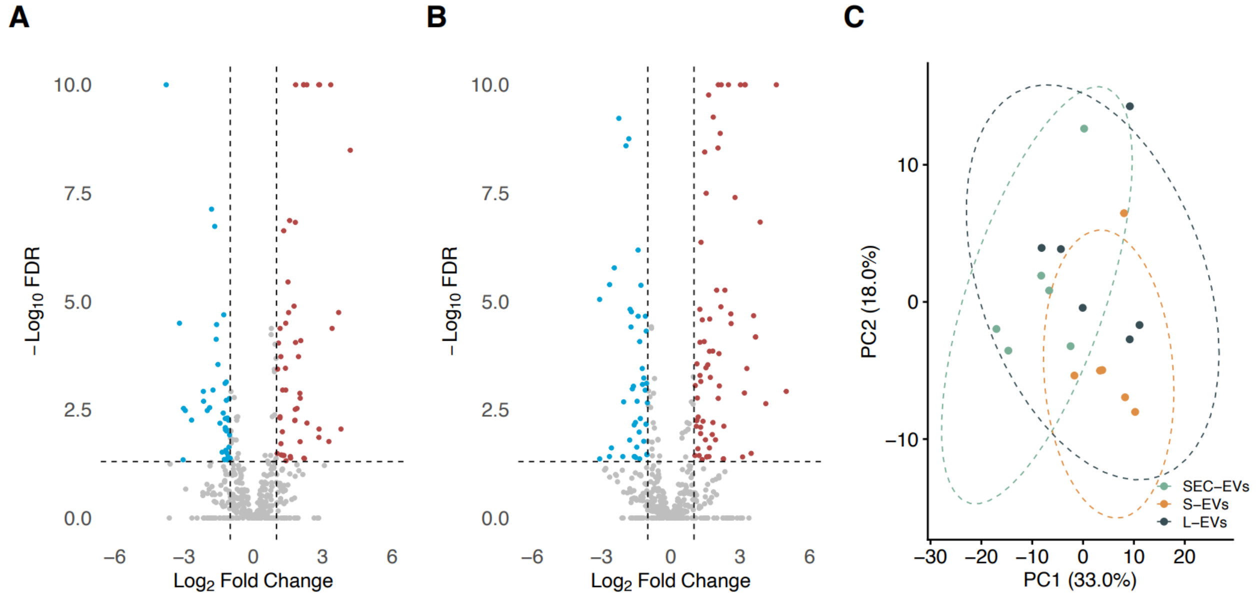

Supplementary Figure 5. Differentially Expressed EV-miRNAs in SEC-EVs compared with UC20K L-EVs and UC100K S-EVs from Baseline DLBCL patients.

(**A**) Volcano plot of differential EV-miRNA expression in SEC-EVs versus UC20K L-EVs. (**B**) Volcano plot of differential EV-miRNA expression in SEC-EVs versus UC100K S-EVs. (**C**) PCA of EV-miRNA expression profiles from SEC-EVs, UC20K L-EVs, and UC100K S-EVs from DLBCL patients. Significantly deregulated transcripts are defined as an absolute log2 fold change > 1 and a Benjamini-Hochberg-adjusted p value (FDR) < 0.05.

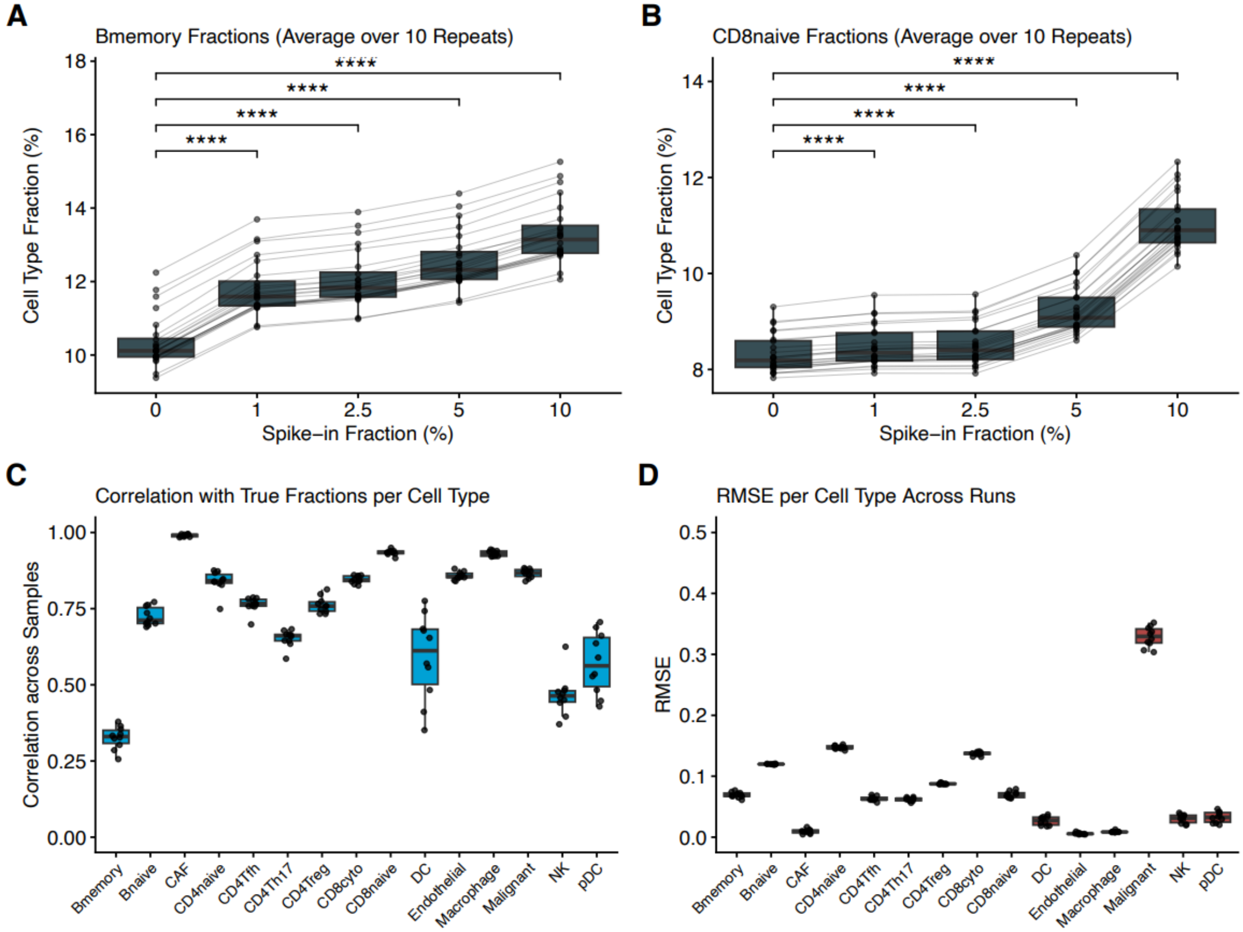

Supplementary Figure 6. Simulation-based validation of EV-mRNA deconvolution performance.

Deconvolution accuracy and robustness were evaluated using *in silico* simulation experiments based on scRNA-seq-derived expression profiles with known ground-truth cell-type proportions. Deconvolution was repeated ten times per condition to assess stability.

(**A**) Memory B cell spike-in experiment. Bulk EV-mRNA profiles were mixed with increasing proportions (0%, 1%, 2.5%, 5%, 10%) of memory B cell scRNA-seq expression. Boxplots show average estimated cell-type fractions across ten repeated deconvolutions, with gray lines connecting individual runs. (**B**) $CD8^+$ cytotoxic T cell spike-in experiment, shown as in (A). Repeated deconvolution robustly captures increasing $CD8^+$ cytotoxic T cell fractions with higher spike-in proportions, despite modest deviations from absolute ground-truth values. (**C**) Pearson correlation coefficients (PCC) between estimated and true cell-type fractions across all cell types and repeated runs. All cell types achieve PCC > 0.25, indicating reliable recovery of relative sample rankings by cell-type proportion. (**D**) Root mean squared error (RMSE) of estimated versus true fractions across cell types. While absolute fraction estimates show variable uncertainty, RMSE values remain low for most immune cell types. Malignant cell fractions exhibit higher RMSE, reflecting increased uncertainty in absolute quantification.

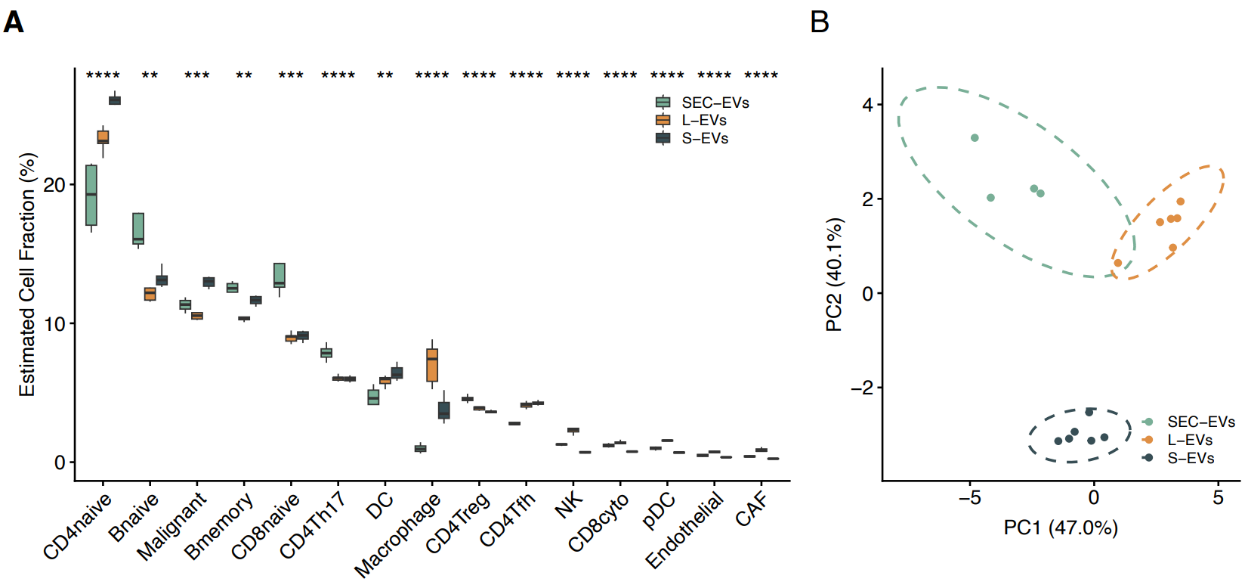

Supplementary Figure 7. Cell-Type Deconvolution of EV subpopulations from Paired Baseline DLBCL Patients.

(**A**) Estimated cell-type fractions of SEC-EVs, UC20K L-EVs, and UC100K S-EVs inferred by Statescope using a single-cell RNA-seq reference encompassing all annotated cell populations. Each box represents the distribution of inferred cell fractions across matched baseline DLBCL samples. Dotted line represents 5%. (**B**) PCA of SEC-EVs, UC100K L-EVs, and UC100K S-EVs based on their deconvoluted cell-fraction profiles, illustrating global compositional differences between EV subpopulations.

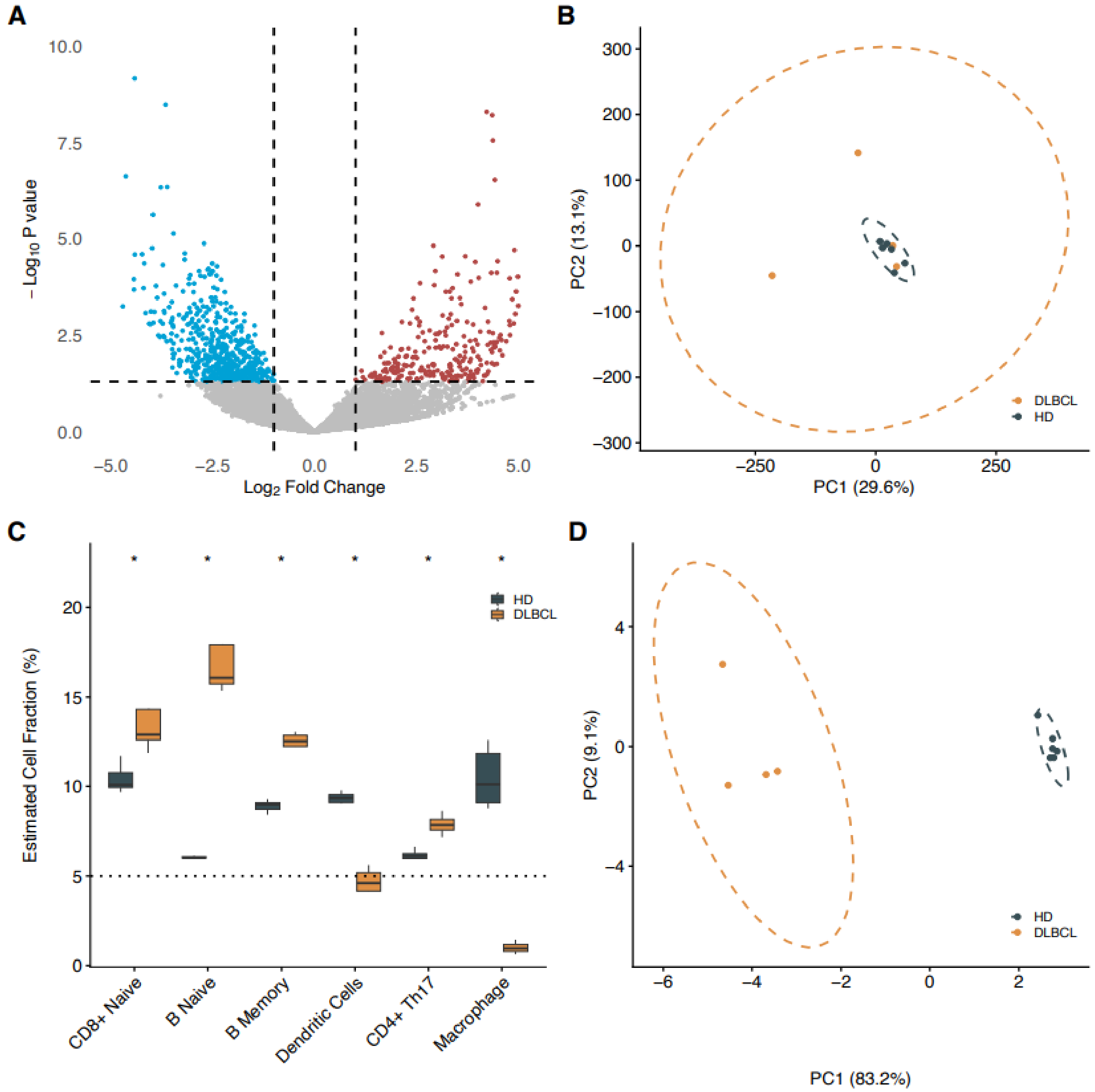

Supplementary Figure 8. SEC-EV mRNA Signatures and Inferred Cell-Type Composition in HD and DLBCL Patients.

(**A**) Volcano plot of differentially expressed SEC-EV mRNAs between DLBCL and HD. Significantly deregulated transcripts are defined as an absolute log2 fold change > 1 and a Benjamini-Hochberg-adjusted p value (FDR) < 0.05. (**B**) PCA of SEC-EV mRNA expression profiles, illustrating global separation between HD and DLBCL patients. (**C**) Comparison of inferred cell-type fractions in SEC-EVs from HD versus DLBCL, showing cell populations with significantly altered representation. (**D**) PCA of SEC-EVs based on Statescope-inferred cell-type fractions, highlighting differences in EV-derived cellular composition between HD and DLBCL.

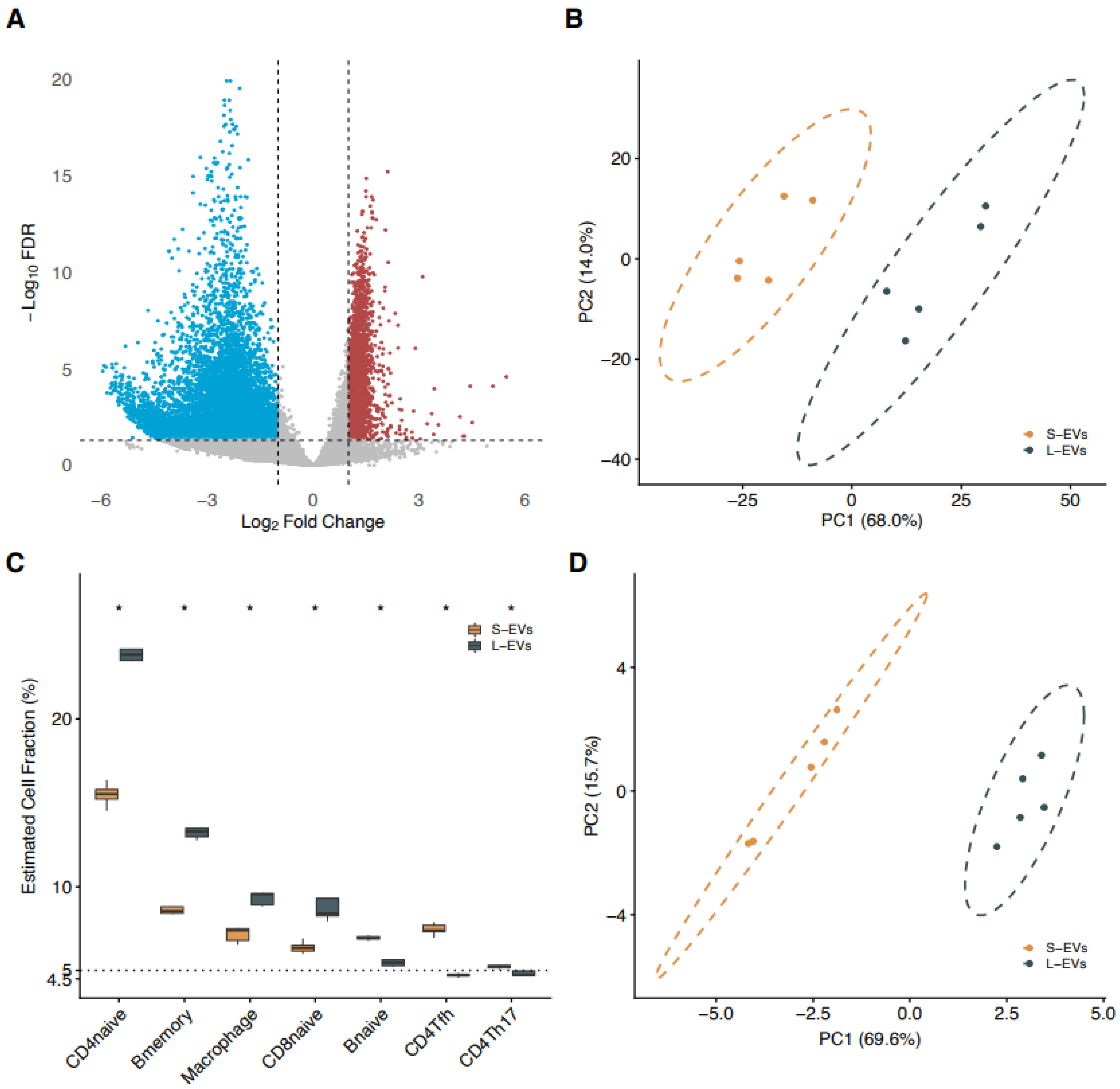

Supplementary Figure 9. Differential Expression and Deconvolution of EV Subpopulations in HD.

(**A**) Volcano plot of differentially expressed genes between HD UC100K S-EVs and UC20K L-EVs. Blue dots represent upregulated mRNAs in UC100K S-EVs and red dots represent upregulated mRNAs in UC20K L-EVs. Significantly deregulated transcripts are defined as an absolute log2 fold change > 1 and a Benjamini-Hochberg-adjusted p value (FDR) < 0.05. (**B**) PCA of HD EV subpopulations based on EV-mRNA expression profiles. (**C**) Statescope-derived cell-type fraction estimates in HD EV subpopulations. (**D**) PCA of HD EV subpopulations based on deconvolved cell-type fractions.

Supplemental Tables

Supplementary Table 1. Patient and Healthy Donor Characteristics.

Abbreviations: DLBCL, diffuse large B-cell lymphoma; HGBL, high-grade B-cell lymphoma; R-CHOP, rituximab, cyclophosphamide, doxorubicin, vincristine, and prednisone; R-CVP, rituximab, cyclophosphamide, vincristine, and prednisone; R-COEP, rituximab, cyclophosphamide, vincristine, etoposide, and prednisone; CMR, complete metabolic response; PMR, partial metabolic response; PMD, progressive metabolic disease.

Supplementary Table 2. SMARTer Mapped EV-RNA Species.

This table summarizes the distribution of RNA species identified by SMARTer Total RNA sequencing across samples. Columns indicate Subject (anonymized subject identifier), Diagnosis, Source (biological material), and Type (EV subpopulation), followed by the relative abundance of RNA species, including mRNA (messenger RNA), scRNA (small cytoplasmic RNA), yRNA, lncRNA (long non-coding RNA), asRNA (antisense RNA), miRNA (microRNA), rRNA (ribosomal RNA), and Others (low-abundance RNA species). UC, ultracentrifugation; SEC, size-exclusion chromatography.

Supplementary Table 3. IsoSeek Mapped EV-RNA Species.

This table summarizes the distribution of RNA species identified by IsoSEEK across samples. Columns indicate Subject (anonymized subject identifier), Diagnosis, Source (biological material), and Type (EV subpopulation), followed by the relative abundance of RNA species, including miRNA (microRNA), yRNA, rRNA (ribosomal RNA), tRNA (transfer RNA), lncRNA (long non-coding RNA), mRNA (messenger RNA), Un-assigned (reads not confidently classified to a known RNA biotype), and Others (low-abundance RNA species). UC, ultracentrifugation; SEC, size-exclusion chromatography.

Supplementary Table 4. Quality Control of SMARTer.

This table summarizes sequencing and alignment metrics for EV total RNA libraries derived from lymphoma cell lines (SU-DHL-4, RI-1, and U2932) and plasma samples. Columns report the EV isolation Method (UC or SEC), Total Reads (raw input reads), Average Input Read Length, Uniquely Mapped % and Multimapped % (fractions of reads aligned to single or multiple genomic locations), Assigned to Genes (reads annotated to gene features), and Detected Genes (genes with ≥ 1 assigned read). UC, ultracentrifugation; SEC, size-exclusion chromatography.

Supplementary Table 5. Quality Control of IsoSeek.

This table summarizes sequencing metrics for EV miRNA libraries generated from lymphoma cell lines (SU-DHL-4, RI-1, and U2932) and plasma samples. Columns report the EV isolation Method (UC or SEC), Adapter dimer % (fraction of reads removed as adapter dimers), Mature miRNA

reads (reads mapped to known mature miRNAs), and Number of miRNAs detected. UC, ultracentrifugation; SEC, size-exclusion chromatography.

Supplementary Table 6. SMARTer EV-mRNA Read Counts.
This table reports gene-level read counts obtained from SMARTer Total RNA sequencing of EV-RNA. Columns list the Gene symbol and annotated Length (bp), followed by raw read counts for each anonymized subject and sample type. For example, DLBCL001_UC20K_LEV represents UC20K L-EV from subject DLBL001. Read counts reflect the number of sequencing reads mapped to each gene per sample prior to differential expression analysis.

Supplementary Table 7. IsoSeek EV-miRNA Read Counts.
This table reports miRNA-level read counts obtained from IsoSeek small RNA sequencing of EV-RNA. Columns list the miRNA identifier, followed by raw read counts for each anonymized subject and sample type. For example, DLBCL001_UC20K_LEV represents UC20K L-EV from subject DLBL001. Read counts represent the number of sequencing reads mapped to each miRNA per sample prior to downstream normalization and differential expression analysis.

Supplementary Table 8. Differentially Expressed EV-mRNAs between UC20K L-EVs and UC100K S-EVs (Reference) in baseline DLBCL patients.
This table lists mRNAs identified as differentially expressed between large extracellular vesicles (L-EVs; UC20K) and small extracellular vesicles (S-EVs; UC100K, reference) in baseline plasma samples from patients with DLBCL. Columns report the gene symbol; log2FC (shrunken log2 fold change, L-EVs vs. S-EVs) with corresponding log2FC_SE and 95% confidence intervals (log2FC_CI_lower, log2FC_CI_upper); log2FC_unshrunk and lfcSE_unshrunk (unshrunk estimates); pvalue and padj (Benjamini-Hochberg-adjusted P value); mean_count_all and median_count_all (overall normalized counts); mean_count_SEVs and mean_count_LEVs (mean normalized counts in S-EVs and L-EVs, respectively); and n_samples_detected (number of samples with detectable expression).

Supplementary Table 9. Differentially expressed miRNAs between UC20K L-EVs and UC100K S-EVs (Reference) in baseline DLBCL patients.
This table lists miRNAs identified as differentially expressed between large extracellular vesicles (L-EVs; UC20K) and small extracellular vesicles (S-EVs; UC100K, reference) in baseline plasma samples from patients with DLBCL. Columns report the miRNA identifier; log2FC (shrunken log2 fold change, L-EVs vs. S-EVs) with corresponding log2FC_SE and 95% confidence intervals (log2FC_CI_lower, log2FC_CI_upper); log2FC_unshrunk and lfcSE_unshrunk (unshrunk estimates); pvalue and padj (Benjamini-Hochberg-adjusted P value); mean_count_all and median_count_all (overall normalized counts); mean_count_SEVs and mean_count_LEVs (mean normalized counts in S-EVs and L-EVs, respectively); and n_samples_detected (number of samples with detectable expression).

Supplementary Table 10. Average Cell Fraction Estimated by Statescope.

This table reports the mean cell-type fractions obtained from ten iterations of Statescope, summarizing the estimated cellular composition across samples. Abbreviations: CD4 naive, naive $CD4^+$ T cells; CD8 cyto, cytotoxic $CD8^+$ T cells; CD4 Treg, regulatory $CD4^+$ T cells; CD8 naive, naive $CD8^+$ T cells; CD4 Tfh, T follicular helper $CD4^+$ T cells; CD4 Th17, T helper 17 $CD4^+$ T cells; B naive, naive B cells; B memory, memory B cells; NK, natural killer cells; pDC, plasmacytoid dendritic cells; Macrophage, macrophages; DC, dendritic cells; Malignant, malignant cells; CAF, cancer-associated fibroblasts; Endothelial, endothelial cells.
